# Documentation of a new hypotrich species in the family Amphisiellidae, *Lamtostyla gui* n. sp. (Protista, Ciliophora) using a multidisciplinary approach

**DOI:** 10.1038/s41598-020-60327-5

**Published:** 2020-02-28

**Authors:** Wanying Liao, Zhiwei Gong, Bing Ni, Xinpeng Fan, Giulio Petroni

**Affiliations:** 1 School of Life Sciences, East China Normal University, Shanghai, 200241 China; 2 Department of Biology, University of Pisa, Via Luca Ghini 13, Pisa, 56126 Italy; 3 School of Physics and Electronic Science, East China Normal University, Shanghai, 200241 China

**Keywords:** Phylogenetics, Taxonomy

## Abstract

An integrated approach considering both morphologic and molecular data is now required to improve biodiversity estimations and provide more robust systematics interpretations in hypotrichs, a highly differentiated group of ciliates. In present study, we document a new hypotrich species, *Lamtostyla gui* n. sp., collected from Chongming wetland, Shanghai, China, based on investigations using living observation, protargol staining, scanning and transmission electron microscopy, and gene sequencing. The new species is mainly recognized by having a short amphisiellid median cirral row composed of four cirri, three frontoventral cirri, three dorsal kinetids, four to eight macronuclear nodules, and small colorless cortical granules distributed as rosettes around dorsal bristles. Transmission electron microscope observation finds the associated microtubules of cirri and pharyngeal discs of *L. gui* are distinct from those in other hypotrichs. Morphogenesis of this species indicates that parental adoral membranelles retained intact or partial renewed is a potential feature to separate *Lamtostyla granulifera-*group and *Lamtostyla lamottei*-group. Phylogenetic analysis based on small subunit ribosomal RNA (rRNA) gene shows that this molecular marker is not useful to resolve phylogenetic relationships of the genus *Lamtostyla*, as well as many other hypotrichous taxa. We additionally characterize the internal transcribed spacers (ITS) region and the almost complete large subunit rRNA, which will be essential for future studies aimed at solving phylogenetic problems of *Lamtostyla*, or even the family Amphisiellidae. As a final remark, the critical screening of GenBank using ITS genes of our organism allows us to recognize a large amount of hypotrichous sequences have been misclassified as fungi. This observation suggests that hypotrichs could be frequently found in fungi-rich environment and overlooked by fungal specialists.

## Introduction

As the highly differentiated group within the phylum Ciliophora, ciliates in the subclass Hypotrichia Stein, 1859 have been drawing a great attention of ciliates taxonomists and revealed to be with indeterminate biodiversity^1–17^. Detailed morphological characteristics and ontogenetic processes are essential for species identification and understanding the systematics in this group^1–4,18–22^. In recent decades, molecular phylogeny studies, especially those based on small subunit ribosomal RNA (SSU rRNA) gene sequence, have helped to answer important questions concerning evolution and phylogenetic relationships of some hypotrichous taxa^10,16,23–29^. Recently, increasing studies have revealed phylogenetic analyses based on multigenes, e.g., SSU rRNA, internal transcribed spacers (ITS, including ITS1, 5.8S, ITS2) region, large subunit ribosomal RNA (LSU rRNA), and alpha tubulin gene could provide more robust interpretations than those using single gene information^30–38^. However, due to the under-sampling of both morphogenetic and molecular data, the phylogenetic relationships of a large number of taxa within this group are still confusing and unresolved. Hence, the integrative taxonomy approach combining multigene-based analyses and detailed optical and electron microscopic observations is suggested to be applied on either known or new species in the Hypotrichia^39–41^.

The genus *Lamtostyla* Buitkamp, 1977 was established and assigned to the family Holostichidae Fauré-Fremiet, 1961 with the shortened ventral row as generic feature^42^. After detailed ontogenetic processes were revealed^21^, *Lamtostyla* was transferred to the family Amphisiellidae Jankowski, 1979 since its shortened ventral row (amphisiellid median cirral row, ACR) originated from cirral anlagen V and VI. This family-level classification has widely been accepted by the following researches^3,43,44^, although, the morphogenetic and molecular information of the type species, *L. lamottei* Buitkamp, 1977 are still lacking. Several species previously assigned to the genus *Amphisiella* Gourret & Roeser, 1888 (type genus of the family Amphisiellidae) were then transferred to *Lamtostyla* based on their short ACR, lower numbers of transverse cirri and dorsal kineties, as well as their terrestrial habitat^3^. To date, 14 species belonging to the genus *Lamtostyla* have been reported: namely, *L. australis* (Blatterer & Foissner, 1988) Petz & Foissner, 1996; *L. decorata* Foissner, Agatha & Berger, 2002; *L. elegans* (Foissner, Agatha & Berger, 2002) Berger, 2008; *L*. *granulifera* Foissner, 1997; *L. islandica* Berger & Foissner, 1988; *L. lamottei*; *L. longa* (Hemberger, 1985) Berger & Foissner, 1988; *L*. *ovalis* Luo *et al*., 2017; *L. perisincirra* (Hemberger, 1985) Berger & Foissner, 1987; *L. procera* (Foissner, Agatha & Berger, 2002) Berger, 2008; *L. quadrinucleata* (Berger & Foissner, 1989) Berger, 2008; *L. raptans* (Hemberger, 1985) Foissner, 1997; *L*. *salina* Dong *et al*., 2016; and *L. vitiphila* (Foissner, 1987) Berger, 2008. Morphological descriptions for most of them are available, but detailed morphogenetic processes have only been reported for *L. australis* and *L. salina*, and SSU rRNA gene sequences are only available in *L*. *ovalis* and *L. salina*^3,17,45^.

In the present study, a previously unknown hypotrich species collected from a marsh wetland at Chongming Island, Shanghai, China was studied using light and electron microscopy and complete ribosomal operon characterization (SSU rRNA, ITS1, 5.8S, ITS2, and LSU rRNA). The organism was revealed as a novel species within the genus *Lamtostyla* and its general morphology, ultrastructure, morphogenesis and SSU rRNA based phylogeny were documented. The complete ribosomal operon sequence we provided will be applied in the future studies, when homologous marker sequences are available for a sufficient number of species, to reveal the proper placement of the described organism within the family Amphisiellidae and to provide a critical evaluation of the monophyly of *Lamtostyla*.

## Results

### General morphology and ciliature

**Figure 1:**
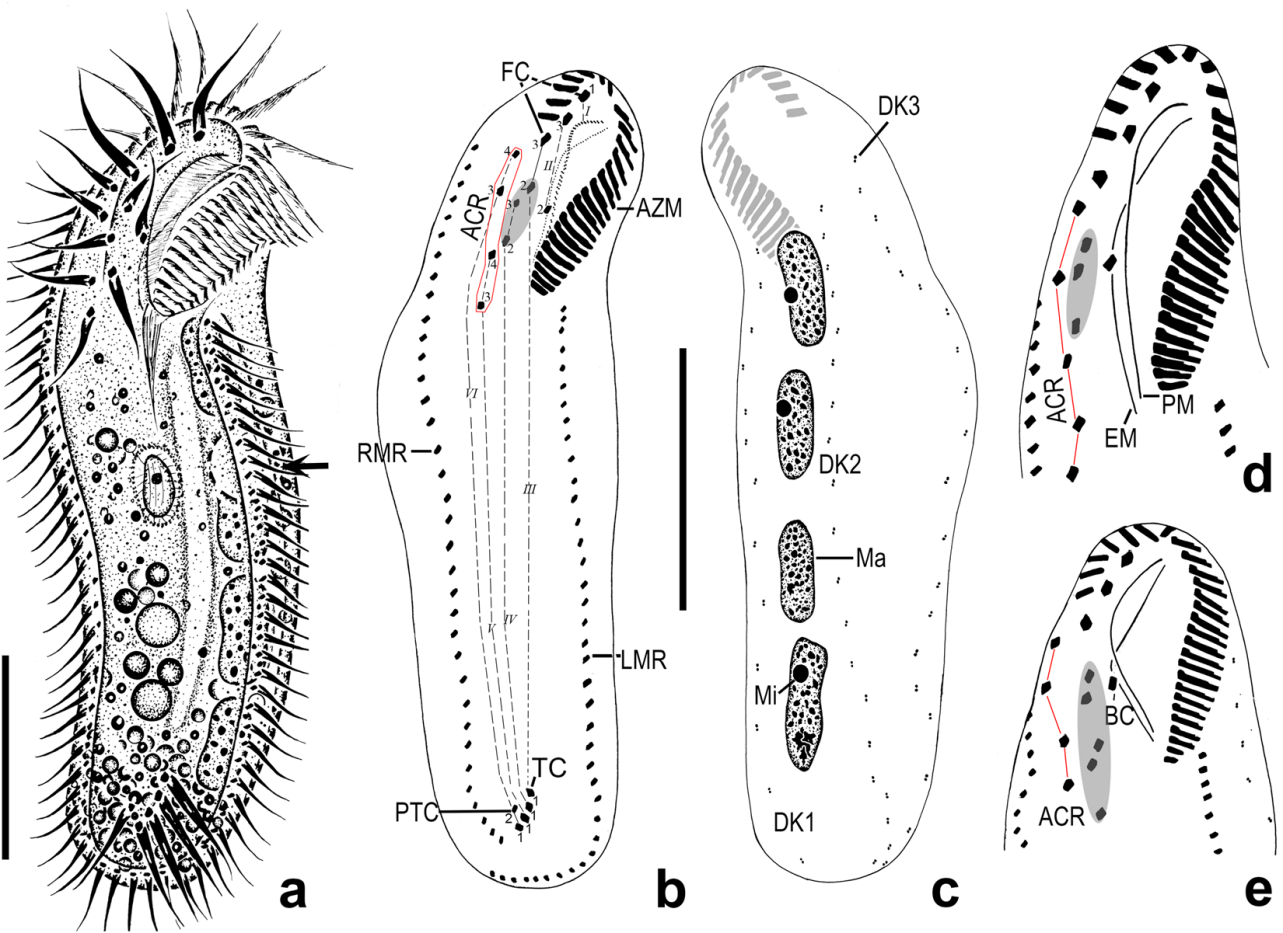
*Lamtostyla gui* n. sp. *in vivo* (**a**) and after protargol staining (**b**–**e**). (**a)** Ventral view of a typical individual, arrow indicates the contractile vacuole. (**b**,**c)** Ventral (**b**) and dorsal (**c**) views of the holotype, showing the infraciliature and nuclear apparatus; the elliptical shadow and the red circle in (**b**) mark the three frontoventral cirri and the ACR, respectivly; cirri originated from the same anlage are connected by dash lines. (**d**,**e)** Variations of frontoventral ciliatures, where the individual (**d**) having five cirri in ACR (red line), and the individual (**e**) having five frontoventral cirri (elliptical shadow). *I–VI* frontoventral transverse cirri anlage I–VI, *ACR* amphisiellid median cirral row, *AZM* adoral zone of membranelle, *BC* buccal cirrus, *DK* dorsal kinety, *EM* endoral membrane, *FC* frontal cirrus, *LMR* left marginal row, *Ma* macronucleus, *Mi* micronucleus, *PM* paroral membrane, *PTC* pretransverse cirrus*, RMR* right marginal row, *TC* transverse cirrus. Scale bar 40 μm.

**Figure 2:**
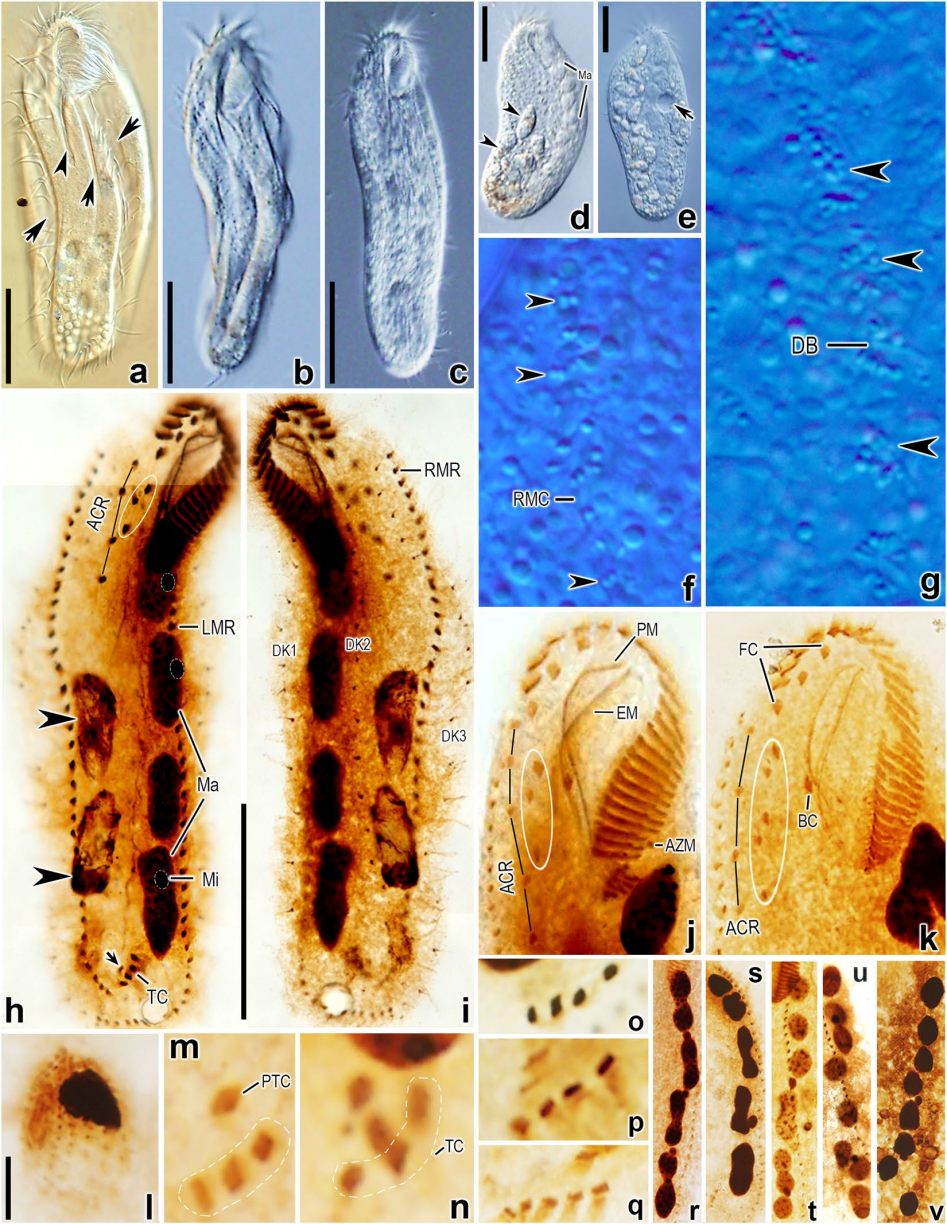
*Lamtostyla gui* n. sp. *in vivo* (**a**–**g**, using DIC microscopy) and after protargol staining (**h**–**v**, using brightfield microscopy). (**a)** Ventral view, arrows show the longitudinal cortical grooves and arrowhead notes the cytopharynx. (**b**,**c)** Ventral views of different cells showing the body shapes. (**d)** Ventral view to show the macronuclei and digested scuticociliates (arrowheads). (**e)** An individual ingested with many scuticociliates, arrow shows the contractile vacuole. (**f**,**g)** The cortical granules (arrowheads) scatter near right marginal cirri on ventral side (**f**) and distribute as rosettes around dorsal bristles on dorsal side (**g**). (**h**,**i)** Ventral (**h**) and dorsal (**i**) views of the holotype specimen, showing the infraciliature and nuclear apparatus; arrow indicates the pretransverse cirrus, arrowheads show the digested scuticociliates and three frontoventral cirri are depicted by circle. (**j**,**k)** Frontoventral ciliatures of two individuals, where the individual (**j**) has five cirri in ACR and three frontoventral cirri (circled); the individual (**k**) has four cirri in ACR and five frontoventral cirri (circled). (**l)** Infraciliature of the digested scuticociliate. (**m**–**q**) Different transverse and pretransverse cirral patterns: one pretransverse cirrus and three transverse cirri in (**m**); two pretransverse cirri and three transverse cirri in (**n**); four transverse cirri in (**o**); two pretransverse cirri and four transverse cirri in (**p**); one pretransverse cirrus and five transverse cirri in (**q**). **(r**–**v)** Variations of macronuclear apparatuses: four binodal nodules in (**r**), three binodal and two ellipsoid nodules in (**s**), two binodal and four globular nodules in (**t**), one binodal and six ellipsoid nodules in (**u**), and eight globular nodules in (**v**). *ACR* amphisiellid median cirral row, *AZM* adoral zone of membranelle, *BC* buccal cirrus, *DB* dorsal bristle, *DK* dorsal kinety, *EM* endoral membrane, *FC* frontal cirrus, *LMR* left marginal row, *Ma* macronucleus, *Mi* micronucleus*, PM* paroral membrane, *PTC* pretransverse cirrus, *RMC* right marginal cirrus, *RMR* right marginal row, *TC* transverse cirrus. Scale bars 40 μm (*a*–*e*,*h*,*i*), 5 μm (**l**).

**Figure 3:**
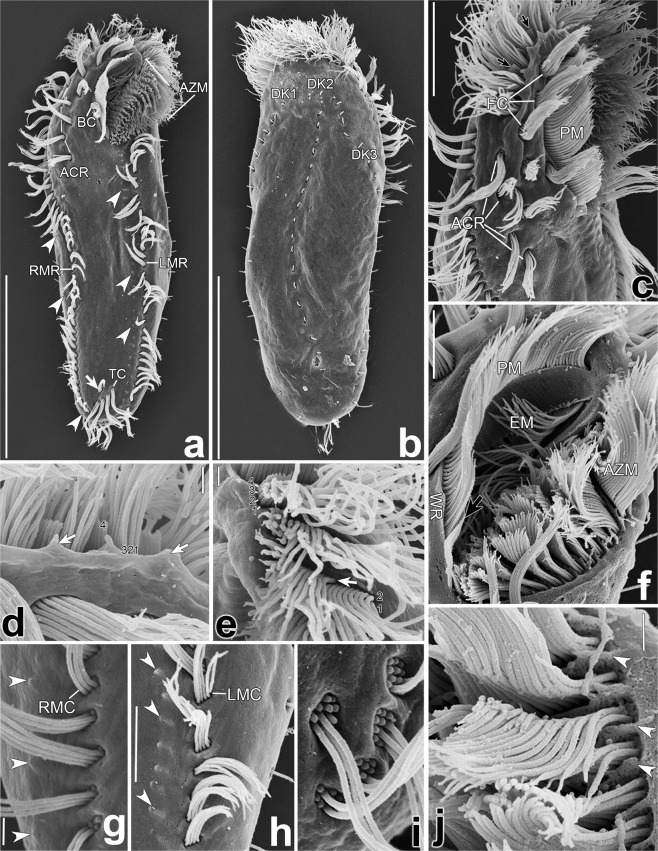
Scanning electron micrographs of *Lamtostyla gui* n. sp. (**a**,**b**) Ventral (**a**) and dorsal (**b**) views of the typical specimens, showing the ciliary pattern; arrow shows the pretransverse cirrus and arrowheads indicate two rows of small cortical protrusions running right of the marginal rows. (**c**) Anterior portion, where three frontal cirri are slightly larger than other cirri, namely, buccal cirrus, four cirri in ACR and three frontoventral cirri; arrows indicate the minute cilia in row 4 of adoral membranelles. (**d**,**e**) Details of collar (**d**) and lapel (**e**) parts of adoral zone, showing the scheme of membranelle composed of four cilia rows marked with number 1–4. Arrows in (**d**) indicate intermembranellar ridges, arrow in (**e**) marks the length of membranellar cilia row 3 is near half of row 2 and 1. (**f**) Details of the buccal region, showing the curved endoral and paroral membranes which composed of monokinetids and dikinetids respectively. Arrow marks the left wall of buccal lip. (**g**,**h**) Details of right (**g**) and left (**h**) marginal rows, revealing that each marginal cirrus was composed of eight kinetosomes and showing some small cortical protrusions (arrowheads) distributed along the right of marginal cirri. (**i**) One pretransverse cirrus and four transverse cirri, where most of ciliary shafts are missing due to the sample preparation. (**j**) Lapel portion of adoral zone showing dorsal view of intermembranellar ridges (arrowheads) between adoral membranelles. *ACR* amphisiellid median cirral row, *AZM* adoral zone of membranelle, *BC* buccal cirrus, *DK* dorsal kinety, *EM* endoral membrane, *FC* frontal cirrus, *LMC* left marginal cirrus, *LMR* left marginal row, *PM* paroral membrane, *RMC* right marginal cirrus, *RMR* right marginal row, *TC* transverse cirrus, *WR* right wall of buccal lip. Scale bars 40 μm (**a**,**b**), 10 μm (**c**), 5 μm (**f**,**h**), and 1 μm (**d**,**e**,**g,j**).

(Table 1, Figs. 1–3) Body size 134–183 × 30–49 μm (151 × 39 μm on average, n = 19) *in vivo*, and 80–185 × 19–67 μm after protargol staining; body flattened dorsoventrally with the ratio of width to thickness up to 2:1, and with length: width ratio between 3:1 to 5:1 *in vivo* (Table 1). Body outline usually elongate elliptical, and sometimes slightly sigmoidal, with anterior portion slightly narrowed and the posterior end broadly rounded (Figs. 1a, 2a–c, 3a,b). Cell highly flexible, but not contractile, with three cortical grooves along left and right marginal rows and ventral meridian in most of individuals (Figs. 1a, 2a–c). Macronuclear nodules highly variable in number and shape: usually four ellipsoidal (Figs. 1a,c, 2h,i) to binodal (Fig. 2r) nodules arranged along near the left cell margin (68 out of 113 analyzed specimens), while other 11 have five (Fig. 2s), 11 have six (Fig. 2t), 9 have seven (Fig. 2u), and 14 have eight (Fig. 2v) globular to binodal nodules (Table 1). Macronuclei 7–27 × 4–13 μm in size after protargol staining (Table 1). One to five (usually three) spherical micronuclei (ca. 3.5 μm across) attached to macronuclear nodules at variable positions (Table 1, Figs. 1c, 2h,r–v). Single contractile vacuole located close to equator near left margin (Figs. 1a, 2e) with interval of contraction 1–2 min. Pellicle thin and soft, with small ellipsoidal (about 0.5 × 0.3 μm) and colorless cortical granules distributing on both ventral and dorsal sides: two or three arranging along marginal cirri (Fig. 2f), while several (about ten) forming a rosette around dorsal bristle (Fig. 2g). Cytoplasm colourless, usually packed with many lipid droplets (1–3 μm across) and food vacuoles (4–20 × 4–9 μm) containing ingested bacteria and small scuticociliates rendered cell opaque and dark at low magnification (Figs. 1a, 2a–e).

**Table 1:** Morphometric characteristics of *Lamtostyla gui* n. sp.

Characters	Min	Max	M	Mean	SD	CV	n
Body length^a^	134	183	152.8	151.3	12.2	8.1	19
Body width^a^	30	49	38.2	39.0	5.0	12.8	19
Ratio of body length to width^a^	3	5	4.0	3.9	0.5	11.7	19
Body length^b^	80	185	130.0	129.8	21.5	16.6	120
Body width^b^	19	67	36.0	36.8	8.5	23.0	107
Anterior body end to proximal end of adoral zone, distance^b^	21	50	33.3	33.3	4.9	14.6	117
Percentage of anterior body end to proximal end of adoral zone, distance to body length^b^	17	41	26.0	26.0	4.0	15.4	116
Anterior body end to proximal end of rightmost ventral cirral row, distance^b^	25	58	40.0	40.4	6.7	16.5	78
Percentage of anterior body end to proximal end of rightmost ventral cirral row, distance to body length^b^	22	42	30.8	30.9	4.1	13.3	77
Transverse cirri to the end of body, distance^b^	3	24	9.8	10.4	3.8	36.9	65
Percentage of transverse cirri to the end of body, distance to body length^b^	2	16	8.1	8.1	2.7	33.1	64
No. of adoral membranelles^b^	20	30	26.0	25.9	1.9	7.3	106
No. of frontal cirri^b^	3	3	3.0	3.0	0.0	0.0	93
No. of buccal cirri^b^	1	1	1.0	1.0	0.0	0.0	93
No. of cirri in ACR^b^	4	5	4.0	4.1	0.3	6.5	93
No. of frontoventral cirri^b^	3	5	3.0	3.1	0.4	11.4	93
No. of left marginal cirri^b^	28	44	36.0	36.6	3.4	9.3	75
No. of right marginal cirri^b^	30	45	38.0	37.7	3.4	9.0	85
No. of transverse cirri^b^	3	5	4.0	4.0	0.3	7.6	87
No. of pre-transverse cirri^b^	0	2	1.0	1.2	0.5	36.8	87
No. of dorsal kineties^b^	3	3	3.0	3.0	0.0	0.0	48
No. of bristles in dorsal kinety 1^c^	12	19	15.0	15.2	1.7	11.1	33
No. of bristles in dorsal kinety 2^c^	15	22	17.0	17.3	1.9	11.1	24
No. of bristles in dorsal kinety 3^c^	15	22	18.0	18.1	1.8	10.2	20
No. of macronuclei^b^	4	8	4.0	5.0	1.5	29.2	113
No. of micronuclei^b^	1	5	3.0	2.7	0.9	32.0	86
Diameter of micronucleus^b^	2	6	3.5	3.5	0.7	20.1	105
Length of macronucleus^b^	7	27	14.0	14.3	5.0	35.3	149
Width of macronucleus^b^	4	13	7.0	7.2	1.7	24.0	149

All data measurements in micrometers. ^a^Data based on living cells, ^b^data based on protargol-stained specimens, ^c^data based on protargol-stained specimens and scanning electron microscopy micrographs. *ACR* amphisiellid median cirral row, *CV* coefficient of variation in percentage, *M* median, *Max* maximum, *Mean* arithmetic mean, *Min* minimum, *n* number of individuals measured, *No*. number, *SD* standard deviation.

Locomotion by slowly crawling on substrate with great flexibility or sometimes rotating around main body axis when swimming. The organism is a very voracious predator: up to 30 cells of scuticociliates (about 20 μm long) found in food vacuoles of one stained specimen (Figs. 1a, 2d,e,h,i,l); moreover, this species also causing the extinction of another hypotrich, *Urosoma salmastra*, in the raw culture by predation on the latter.

Adoral zone about 26% of body length and spoon-like, comprising 20–30 adoral membranelles with cilia up to 20 μm long, and bases of largest membranelles about 12 μm wide *in vivo* (Table 1, Figs. 1a–e, 2a–e,h–k, 3a). Membranelles comprising four ciliary rows in unequal length: row 4 shortest with only three to five minute cilia, row 3 about half length of row 2 and 1 (Figs. 1b,d,e, 2j,k, 3c–e). In scanning electron microscopy (SEM) prepared specimens, adjacent membranelles separated by intermembranellar ridges (Fig. 3d,e,j) and no lateral membranellar cilia found (Fig. 3a,e,f). Buccal cavity deep and narrow, with curved buccal lip as its right margin, partially covering proximal portion of adoral zone (Fig. 3a,f). Paroral membrane consisting of two rows of cilia, extending along buccal lip; left wall of buccal lip higher than right wall at its posterior end (Figs. 1b, 3a,c,f). Endoral membrane comprised of single row, distinctly curved, extending diagonally on dorsal wall of buccal cavity (Figs. 1b, 3a,f). Endoral and paroral membranes nearly equal in length, about 30 μm after protargol staining, and optically intersecting with each other (Figs. 1a,b,d,e, 2h–k). Pharyngeal fibres prominent *in vivo*, about 20 μm long, extending obliquely backwards (Figs. 1a, 2a).

Invariable three slightly enlarged frontal cirri, cilia about 15 μm long *in vivo*, arranging in oblique row, with rightmost cirrus behind distal end of adoral zone of membranelles; single buccal cirrus, behind middle part of paroral membrane (Table 1, Figs. 1a,b,d,e, 2h,j,k, 3a,c). ACR composed of four or five cirri, commencing close to the rightmost frontal cirrus and terminating on average at 31% of body length with three to five frontoventral cirri on its left (Table 1, Figs. 1a,b,d,e, 2h,j,k, 3a,c). Of 93 investigated specimens, 82 cells possessed four cirri in ACR and three frontoventral cirri (Figs. 1a,b, 2h, 3a,c); five cells possessed five cirri in ACR and three frontoventral cirri (Figs. 1d, 2j); four cells possessed four cirri in ACR and five frontoventral cirri (Figs. 1e, 2k); two cells possessed four cirri in ACR and four frontoventral cirri (Table1). Usually one pretransverse cirrus (PTC) and four transverse cirri (TC), that is the common “1 + 4” pattern (57 out of 87 specimens, Figs. 1a,b, 2a,h, 3a,i). Variations occurred as “2 + 4” pattern (21 out of 87 specimens, Fig. 2p), “1 + 3” pattern (4 out of 87 specimens, Fig. 2m), “1 + 5” pattern (3 out of 87 specimens, Fig. 2q); “2 + 3” pattern (1 out of 87 specimens, Fig. 2n), and “0 + 4” (1 out of 87 specimens, Fig. 2o) (Table 1). Cilia in PTC/TC about 20 μm long *in vivo* and projected beyond rear body end (Figs. 1a, 2a, 3a). 28–44 left and 30–45 right marginal cirri, each composed eight kinetosomes arranged in two rows, with cilia about 14 μm long *in vivo* (Table 1; Figs. 1a,b, 2a,h,i, 3a,g,h). Right marginal row commencing at level of rightmost frontal cirrus or anteriormost cirrus of ACR, and terminating posterior to transverse cirri; left marginal row starting posterior to adoral zone and extending along margin of posterior body end, so that marginal rows distinctly separated posteriorly (Figs. 1a,b, 2a,h,i, 3a). In addition, two rows of small protrusions extending along the right sides of marginal rows shown on SEM (Fig. 3a,g,h). Three bipolar dorsal kineties with about 15, 17, 18 dikinetids in each rows; bristles located on anterior kinetosome of each dikinetid and about 3 μm long *in vivo* (Table 1, Figs. 1c, 2g,i, 3b). No caudal cirri.

**Table 2:** Number of new cirri formed from the frontoventral transverse cirri anlagen I–VI in the daughter cells of dividing individuals of *Lamtostyla gui* n. sp.

Anlage	Min	Max	M	Mean	SD	CV	n
I	1	1	1	1.0	0.0	0.0	32
II	2	2	2	2.0	0.0	0.0	32
III	2	4	3	3.0	0.3	8.5	32
IV	3	4	3	3.1	0.3	9.6	32
V	3	4	3	3.2	0.4	11.7	32
VI	3	5	4	4.0	0.4	9.0	32

*CV* coefficient of variation in percentage, *M* median, *Max* maximum, *Mean* arithmetic mean, *Min* minimum, *n* number of individuals measured, *SD* standard deviation.

### Morphogenesis

#### Stomatogenesis

**Figure 4:**
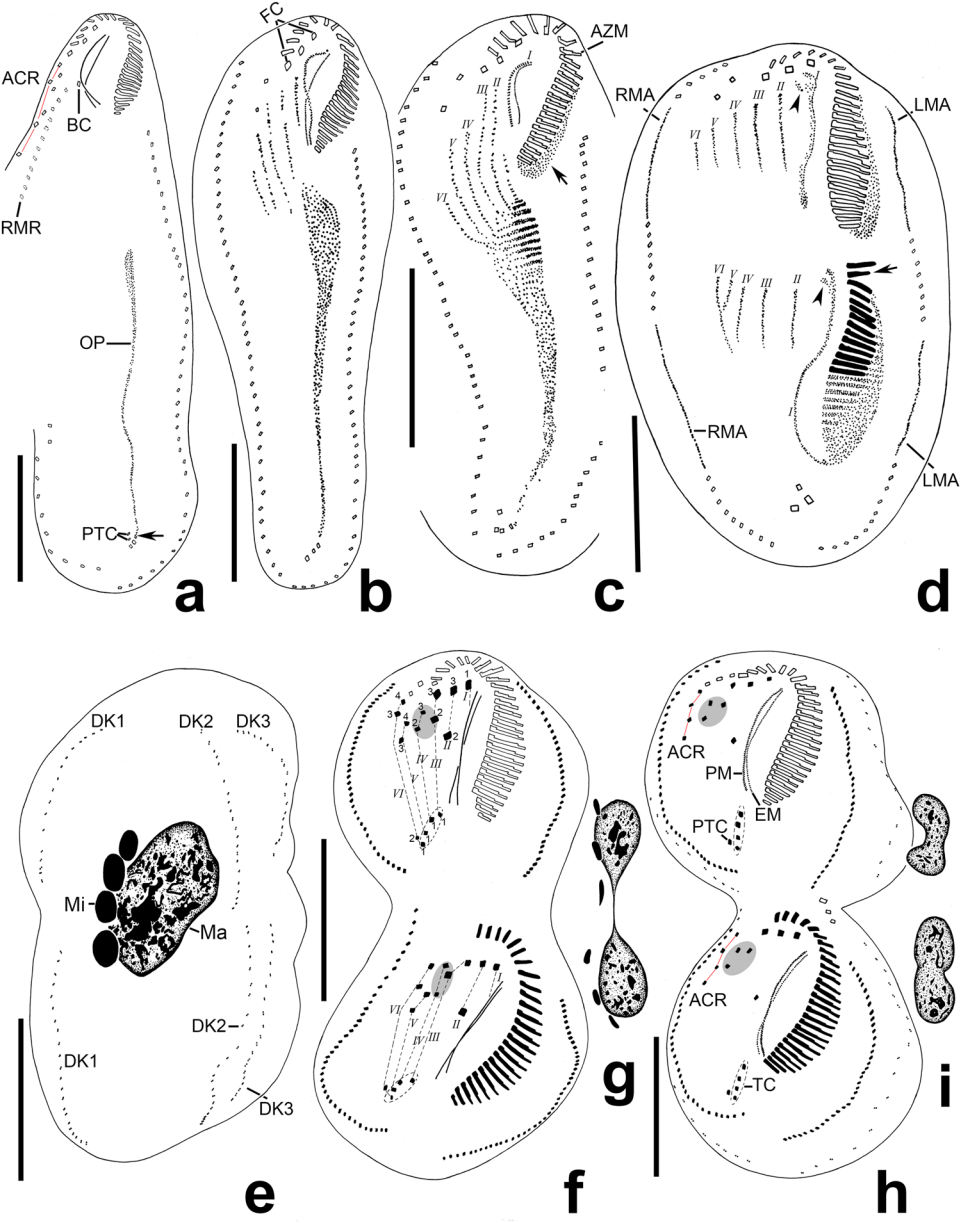
Morphogenesis of *Lamtostyla gui* n. sp. after protargol staining. (**a**) Ventral view of an early divider, to show the formation of oral primordium in the opisthe; arrow marks the origin of oral primordium may from the dedifferentiation of the leftmost one or two transverse cirri. The cirral row (dashed square) left of the ACR is anterior part of the right marginal row extended to the dorsal side. (**b**,**c**) Ventral views of early dividers, to indicate the formation of frontoventral transverse cirri anlagen and the developing of oral primordium; arrow in (**c**) marks the reorganization of the proximal membranelles of adoral zone in the proter. (**d**) Ventral view of a middle divider showing two sets of six frontoventral-transverse cirral anlagen, left and right marginal row anlagen in the proter and opisthe. Arrow marks the oral primordium differentiating to new membranelles and arrowheads note the undulating membranes anlage giving rise to the leftmost frontal cirrus in both proter and opisthe. (**e)** Dorsal view of a late divider, to show nuclear apparatus and dorsal kineties. (**f**–**i)** Ventral views (**f**,**h**) and nuclear apparatuses (**g**,**i**) of two later dividers with two sets of six frontoventral transverse cirri anlagen; cirri originated from the same anlage are connected by dash lines; shadows and dashed ellipses in (**f**–**h**) respectively mark the three frontoventral cirri and four transverse cirri. *I–VI* frontoventral cirri transverse anlage I–VI, *ACR* amphisiellid median cirral row, *AZM* adoral zone of membranelle, *BC* buccal cirrus, *DK* dorsal kinety, *EM* endoral membrane, *FC* frontal cirrus, *LMA* left marginal row anlage, *Ma* macronucleus, *Mi* micronucleus, *OP* oral primordium, *PM* paroral membrane, *PTC* pretransverse cirrus, *RMA* right marginal row anlage, *RMR* right marginal row, *TC* transverse cirrus. Scale bars 40 μm.

**Figure 5:**
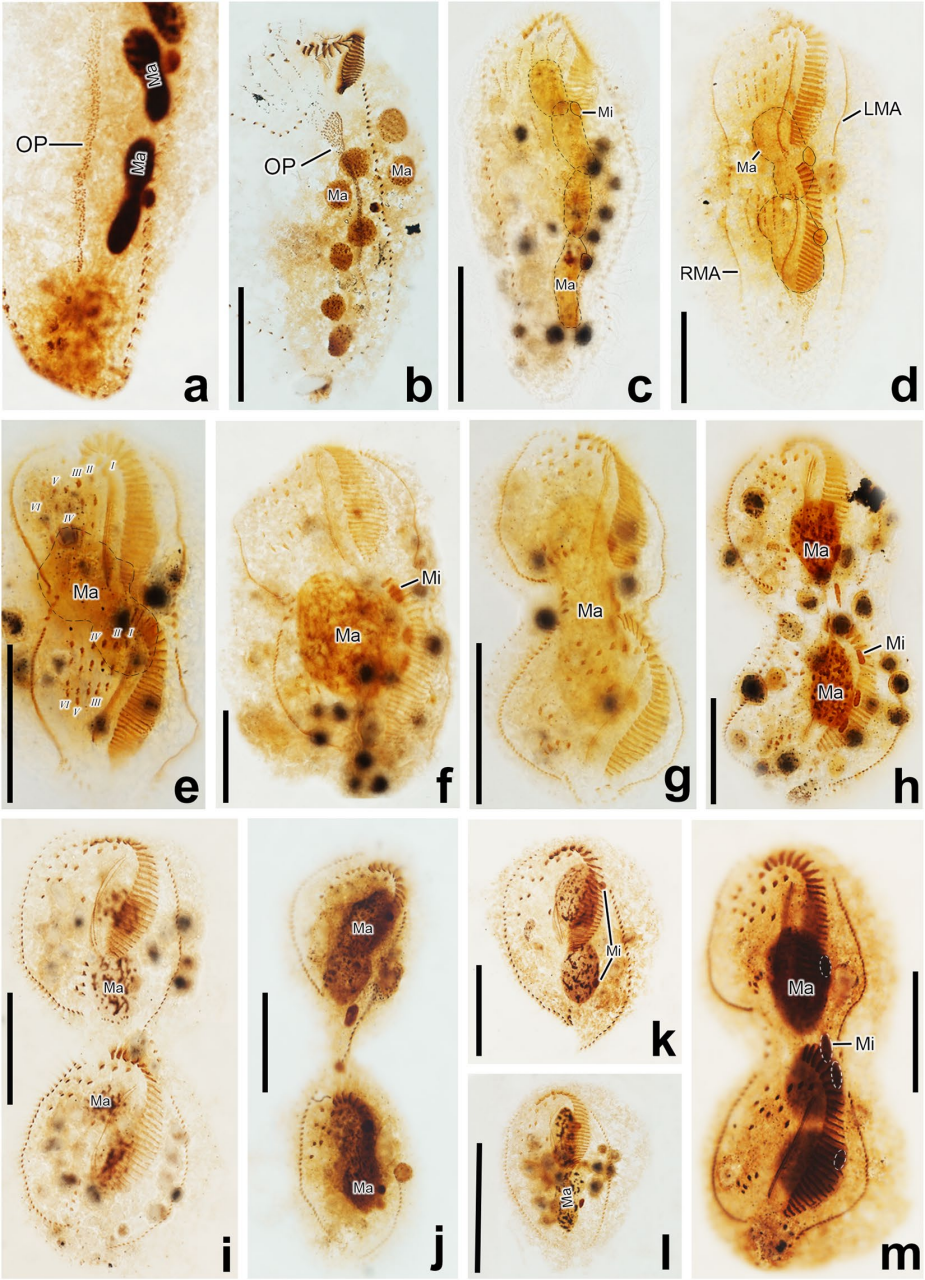
Photomicrographs of *Lamtostyla gui* n. sp. during binary division after protargol staining. (**a)** Ventral view of an early divider to show the formation of oral primordium originated from the leftmost transverse cirrus. (**b**–**e**) Ventral views of some early to middle dividers to show the formation and fragmentation of the six frontoventral-transverse cirral anlagen in the proter and opisthe. (**f**,**g**) Ventral views of two middle stage dividers to show the fusion and division of the macronucleus. (**h**) Ventral view of a later stage divider to show that the single macronuclear nodule divides into two ellipsoidal macronuclear nodules. (**i**,**j**) Ventral views of two later dividers at the end of cytokinesis process, showing that each daughter cell has two separate (**i**) or overlapped (**j**) ellipsoidal macronuclear nodules. (**k**,**l**) Two proters after cytokinesis, with two ellipsoidal (**k**) or binodal (**l**) macronuclear nodules and two micronuclei. (**m**) Three ellipsoidal macronuclear nodules and four micronuclei distributed in a later stage divider. *I–VI* frontoventral transverse cirri anlage I–VI, *LMA* left marginal row anlage, *Ma* macronucleus, *Mi* micronucleus, *OP* oral primordium, *RMA* right marginal row anlage. Scale bars 40 μm.

**Figure 6:**
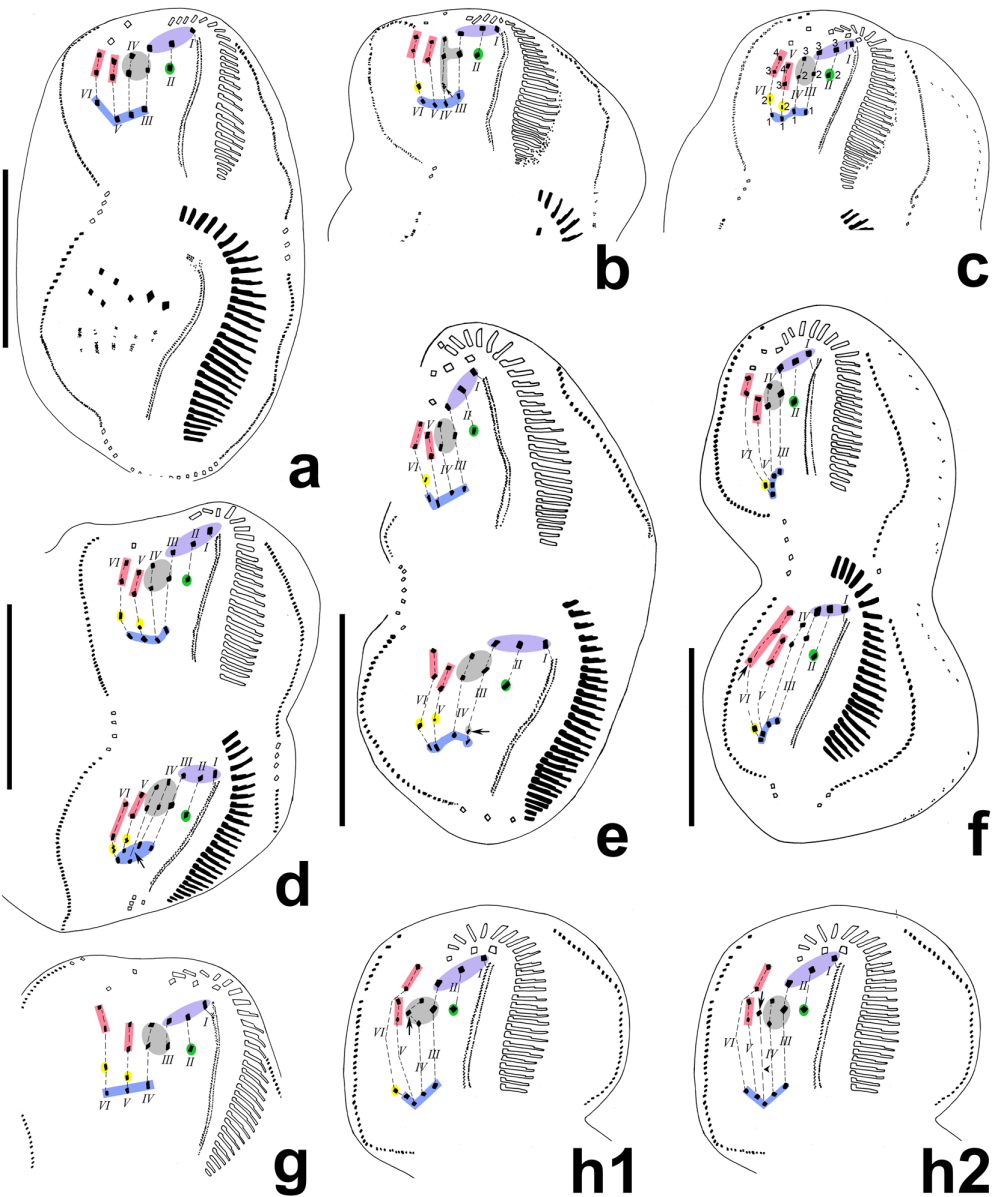
Middle or late stages of unusual morphogenesis patterns in *Lamtostyla gui* n. sp. after protargol staining. (**a**) Ventral view of a middle divider with four transverse cirri in the proter. (**b,c**) Ventral views of two late dividers with only the proters shown; arrow in (**b**) indicates the extra frontoventral cirrus produced by anlage IV; a proter with two pretransverse cirri (cirrus V/2 and VI/2) and four transverse cirri is shown on (**c**); (**d**) Ventral view of a late divider whose proter follows the normal six frontoventral transverse cirri anlagen pattern, whereas, having an additional anlage (arrow) between anlage IV and V in the opisthe. (**e)** Ventral view of a late divider where the proter follows the common morphogenesis pattern, but its opisthe has one extra anlage III-originated cirrus (arrow). (**f**) Ventral view of another late divider whose cirral alignment is normal in the proter but with one extra anlage VI-originated cirrus (arrow) in the opisthe, causing five cirri in ACR. (**g**) Ventral view of one proter with two pretransverse cirri and three transverse cirri. (**h1**, **2**) Two interpretations on the origination of a supernumerary cirrus (arrow) in the same proter: it may come from anlage IV (shown in **h1**) or from one additional anlage (arrowhead) between anlage IV and V (shown in **h2**). Frontal cirrus highlights in purple, buccal cirrus in green, frontoventral cirrus in grey, ACR in red, pretransverse cirrus in yellow, and transverse cirrus in blue. Cirri originated from the same anlage are connected by dash lines. *I–VI* frontoventral transverse cirri anlage I–VI. Scale bars 40 μm.

(Tables 2, 3, Figs. 4–6) In the opisthe, stomatogenesis commences with the formation of an oral primordium which originates from the dedifferentiation of the leftmost one or two transverse cirri (Figs. 4a–c, 5a). The oral primordium enlarges into a long field with broadened anterior end and then the new adoral membranelles begin to organize at the anterior end of the primordium (Figs. 4b–d, 5b–d). At the same time, the undulating membranes anlagen (UMA), i.e., the frontoventral transverse cirri (FVT) anlage I, is formed to the right of the oral primordium (Figs. 4d, 5d). Later, the leftmost frontal cirrus is generated from the anterior end of the UMA (Figs. 4d, 5d). In the later stage, the anterior end of the newly built adoral zone bends to the right and the differentiation of membranelles is almost completed; meanwhile, the UMA splits longitudinally into two streaks which give rise to the paroral and endoral membrane, respectively (Figs. 4f,h, 5e–g,i).

**Table 3:** Variations in number of cirri produced by each anlage during morphogenesis explaining the inconsistency of ventral cirral patten in interphase specimen in *Lamtostyla gui* n. sp.

FVT anlagen	No. of cirri produced in each anlage							→	Corresponding ventral cirral pattern (No.)						Cirral variation position	Referring figures
	I	II	III	IV	*	V	VI	→	FC	BC	ACR^a^	FVC	PTC	TC		
6 FVT anlagen	1	2	3	3	/	3	4	→	3	1	4	3	1	4	Most common pattern	Figs. 4f,h, 6e,f (proters)
	1	2	3	3	/	3	**3**	→	3	1	4	3	**0**	4	PTC	Fig. 6a (proter)
	1	2	3	3	/	**4**	4	→	3	1	4	3	**2**	4	PTC	Fig. 6c,d (proter)
	1	2	**2**	3	/	**4**	4	→	3	1	4	3	**2**	**3**	PTC & TC	Fig. 6g
	1	2	3	**4**	/	3	4	→	3	1	4	**4**	1	4	FVC	Fig. 6b,h1
	1	2	**4**	3	/	**4**	4	→	3	1	4	**4**	**2**	4	FVC & PTC	Fig. 6e (opisthe)
	1	2	3	3	/	3	**5**	→	3	1	**5**	3	1	4	ACR	Fig. 6f (opisthe)
6 FVT anlagen plus an additional anlage	1	2	3	3	**2**	3	**3**	→	3	1	4	**4**	**0**	**5**	FVC, PTC & TC	Fig. 6h2
	1	2	3	3	**3**	**4**	4	→	3	1	4	**5**	**2**	**5**	FVC, PTC & TC	Fig. 6d (opisthe)

^a^cirri in ACR, *the additional anlage between anlage IV and V, / not applied, *ACR* amphisiellid median cirral row, *BC* buccal cirrus, *FC* frontal cirrus, *FVC* frontoventral cirrus, *FVT* frontoventral transverse cirri, *No*. number, *PTC* pretransverse cirrus, *TC* transverse cirrus. Variations (comparing with the most common pattern) in the number of cirri are shown in bold.

In the proter, parental adoral membranelles are mostly retained, with only those in the posterior end renewed *in situ* (Figs. 4c,d, 5c–f). The UMA is formed from the dedifferentiation of the parental undulating membranes (Figs. 4b–d, 5b,c). In subsequent stages, the development of the UMA follows a similar pattern to that in the opisthe (Figs. 4f,h, 5d–i).

#### Development of the frontoventral transverse cirri

In the early stage, buccal cirrus (II/2), three frontoventral cirri (cirrus III/2, IV/2, IV/3) and the posterior two cirri of ACR (cirrus V/3, 4) dedifferentiate into small groups of kinetosomes when the oral primordium of the opisthe begins to elongate (Fig. 4b). Later, these long anlagen connect with the anterior end of the oral primordium, forming five thread-like primordia with anlage I of both proter and opisthe disconnected (Figs. 4c, 5b). Then, the primordia separate very soon, usually two sets of six FVT anlagen (I–VI) of the proter and opisthe are formed (Figs. 4d, 5c). Subsequently, these cirral anlagen develop independently in both dividing parts. Each anlage begins to segment into new cirri (Figs. 4f,h, 5d–j,m): as is usual, anlage I generates left frontal cirrus (cirrus I/1) and undulating membranes; anlage II produces buccal cirrus (cirrus II/2) and middle frontal cirrus (cirrus II/3); anlage III forms one transverse cirrus (cirrus III/1), one frontoventral cirrus (cirrus III/2), and rightmost frontal cirrus (cirrus III/3); anlage IV forms one transverse cirrus (cirrus IV/1) and two frontoventral cirri (cirrus IV/2, 3); anlage V produces one transverse cirrus (cirrus V/1) and the posterior two cirri of ACR (cirrus V/3, 4); anlage VI forms one transverse cirrus (cirrus VI/1), one pretransverse cirrus (cirrus VI/2) and the anterior two cirri of ACR (cirrus VI/3, 4) (Tables 2, 3, Fig. 4f,h). Occasionally, the anlagen III–VI form more or less cirri compared with most common pattern (Table 2), from which we can deduce reasonable explanations for some variations on the number of cirri in frontoventral and transverse regions in interphasic specimens (Table 3, Fig. 6a–h2). Besides,one additional anlage is sometimes present between IV and V (Fig. 6d,h2) which could contribute one more TC and one (Fig. 6h2) or two (Fig. 6d) more frontoventral cirri causing cells with five TC and four or five frontoventral cirri. As usual, the parental three frontal cirri and cirrus VI/3, 4 (anterior two cirri in ACR) are never involved in the FVT-anlagen formation and remain unchanged even in the very late dividers (Figs. 4f,h, 5g–j,m, 6a–h2), indicating that they may be resorbed after cell division.

#### Development of marginal rows and dorsal kineties

The marginal anlagen are formed at two levels within the parental marginal rows, i.e., near the anterior end, and below the mid-body; they then stretch posteriad and gradually replace the parental rows (Figs. 4d, 5d–g, 6a). Unfortunately, the early and middle stages of dorsal kinety anlagen formation are not observed, and only a very late stage shows that three dorsal kineties are newly formed in both dividers (Fig. 4e). Dorsomarginal rows, dorsal kinety fragmentation and caudal cirri are lacking.

#### Division of nuclear apparatus

The nuclear apparatus divides in a conventional manner for hypotrichous ciliates^3^, that is, four to eight macronuclear nodules fuse to form a single mass during the mid-divisional stage (Figs. 4e, 5c–f) and subsequently divides into two ellipsoidal nodules (Figs. 4g, 5g,h). Then each nodule divides again before cytokinesis (Figs. 4i, 5i,j). Two ellipsoidal (Fig. 5k) to binodal (Fig. 5l) macronuclear nodules could often be found in the newly separated daughter cells. Hence, after the cytokinesis, the division of macronuclear nodules may still proceed in daughter cells to form four or more macronuclear nodules as shown in the trophic stage. Sometimes, three ellipsoidal macronuclear nodules occur in a later divider (Fig. 5m), which may result from the asynchronization of nuclear division in the proter and opisthe. Micronuclei were observed to divide mitotically (Figs. 4g, 5h).

### Transmission electron microscope observations

**Figure 7:**
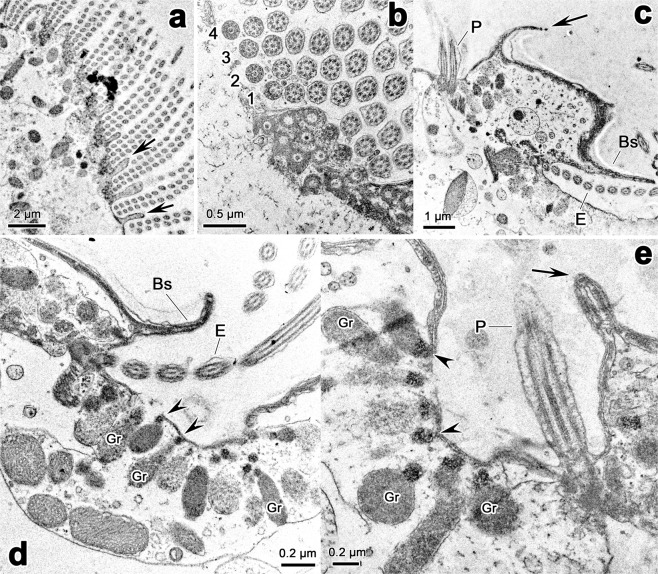
Transmission electron micrographs of the buccal area of *Lamtostyla gui* n. sp. (**a,b**) Adoral zone of membranelles, arrows in (**a**) show the intermembranellar ridges, 1–4 in (**b**) show the four cilia rows within a membranelle, among them, row 4 only contains three kinetosomes. (**c**–**e**) Different sections through buccal lip (arrows), buccal seal, paroral and endoral membranes and nearby cortex. Arrowheads show the small high electron-dense anterior part of the inclusion of cortical granules, which can be easily recognized when they located near paroral and endoral membranes. *Bs* buccal seal, *E* endoral membrane, *Gr* cortical granule, *P* paroral membrane.

**Figure 8:**
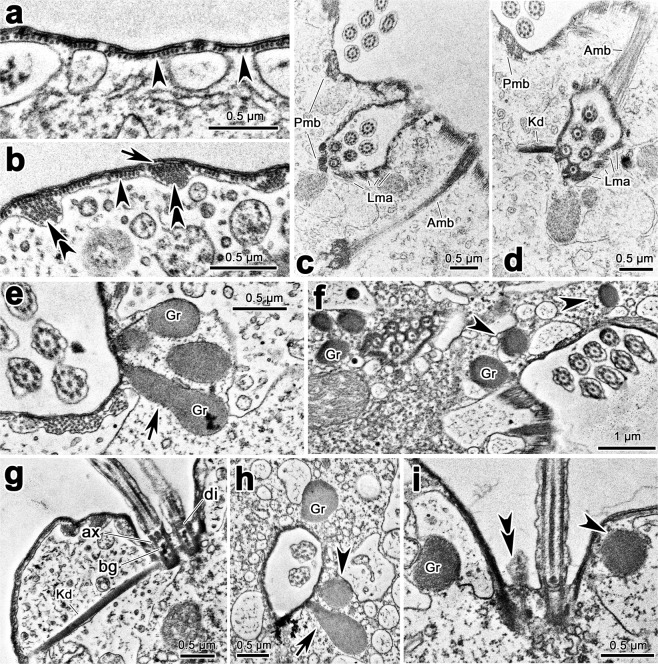
Transmission electron micrographs of the cortex of *Lamtostyla gui* n. sp. (**a**,**b**) Pellicle, arrow and arrowheads show the pellicular alveolus and the subpellicular microtubules, respectively; double-arrowheads depict the thickened microtubule bundles. (**c,d**) Different sections through cirri and the nearby cortex, to show the associated microtubules of cirri, i. e., anterior microtubule bundle, posterior microtubule bundle and the linear microtubular arrays, which are connected with rampart; while, kinetodesmal fiber is connected with kinetosomes. (**e,f**) Sections through cirri, to show the spherical (arrowheads in **f**) and sometimes teardrop-shaped (arrow in **e**) cortical granules arranging in the vicinity of the kinetosomes of cirri. (**g**) Section through a cirrus, showing the kinetodesmal fiber connecting with the kinetosomes of a cirrus, and the fine structure of the kinetosomes consisting of axosome, basal plate, and basal granule. (**h**,**i**) Transection (**h**) and longitudinal (**i**) sections of a dorsal bristle unit where both spherical shaped (arrowheads) and teardrop-shaped (arrow in **h**) cortical granules are present; double-arrowheads in (**i**) notes the barren kinetosome. *Amb*, anterior microtubular bundle, *ax* axoneme, *bg* basal granule, *di* diaphragm, *Gr* cortical granule, *Kd* kinetodesmal fiber, *Lma* linear microtubular arrays, *Pmb* posterior microtubular bundle.

**Figure 9:**
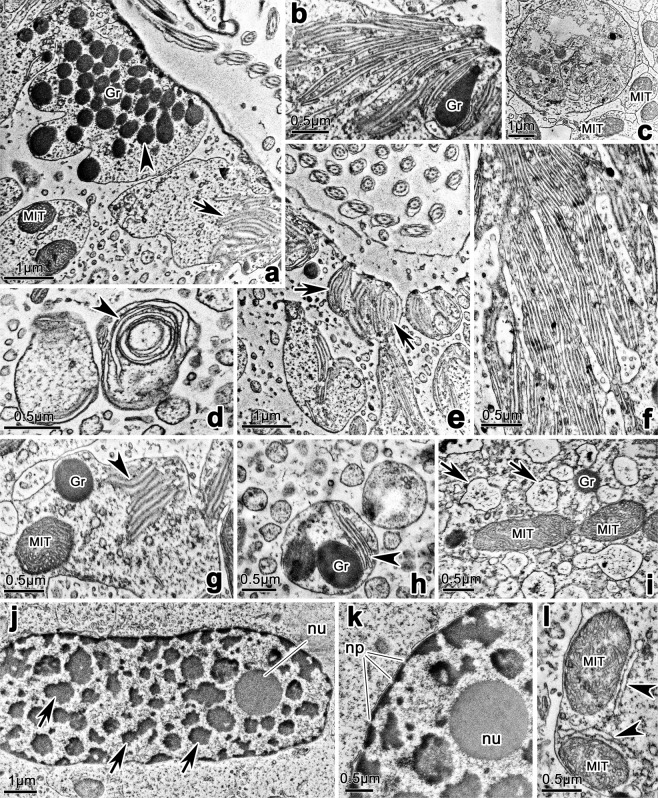
Transmission electron micrographs focusing on the buccal area, cytoplasm, and macronucleus of *Lamtostyla gui* n. sp. (**a**,**e)** Two diagonally spliced views showing the aggregation of cortical granules (arrowheads) and flattened saccules (arrows) beneath the pellicle of the buccal area. (**b**,**f**) The flattened saccules in the cytoplasm near the pellicle of buccal area exist in laminar form, and teardrop-shaped cortical granule occasionally presents among the flattened vesicles. (**c**) A food vacuole with some mitochondria around it. (**d**,**g**,**h**) The flattened saccules (arrowheads) distributed in deeper cytoplasm near the buccal area, are usually curly (**d**) or shortened (**g**,**h**); and sometimes are encased in cytoplasmic vesicles with cortical granules inside (**h**). (**i**) The cytoplasm far away from the buccal area contains mitochondria, cortical granules, and numerous cytoplasmic vesicles (arrows). (**j**) A macronucleus, showing the chromatin bodies (arrows) and nucleolus. (**k**) Part of a macronucleus, showing the discontinuous nuclear envelope with nuclear pores. (**l**) Endoplasmic reticula (arrowheads) which are usually present close to mitochondria. *Gr* cortical granule, *MIT* mitochondrion, *np* nuclear pore, *nu* nucleolus.

(Figs. 7–9) Adoral membranelles were separated by intermembranellar ridges which measured approximately 2 μm at their highest (Fig. 7a). Each membranelle comprised four rows of kinetosomes, with row 4 containing only three kinetosomes in the available sections (Fig. 7b), which is consistent with SEM observation. The left wall of buccal lip and buccal seal were found in a protrusion between paroral and endoral membranes; and they were either straight or curved at their distal end in different sections (Fig. 7c–e). The pellicle consisted of plasma membrane and alveoli; the alveoli were relatively flat, and sometimes hard to be noticed (Fig. 8a,b). A single layer of subpellicular microtubules was beneath the pellicle in most parts of cell (Fig. 8a,b); and sometimes thickened microtubular bundles were also present, especially in the vicinity of cirri, which were possibly the associated microtubules of cirri (Fig. 8b). The associated microtubules of left marginal cirri were observed in details. The anterior microtubular bundles (Amb) and the posterior microtubular bundles (Pmb) connected with the rampart in the two shorter sides of cirri base; the linear microtubular arrays (Lma) strengthened the rampart in the left longer side of cirri base; and the kinetodesmal fiber connected directly with kinetosomes of cirri (Fig. 8c,d). Each kinetosomes consisted of axosome, basal plate and basal granule (Fig. 8g).

The cortical granules were composed of a membrane and the internal inclusion (Figs. 7d,e, 8e,f,h,i, 9a,b,g–i). Most of them were round to oval and measured 0.3–0.6 μm at their widest (Figs. 7d,e, 8e,f,h, 9a,g–i); while they were elongated and teardrop-shaped (up to 1.2 μm long) in some sections and more often present near the buccal area (Figs. 7d,e, 8e,h, 9b). These granules were ordinarily in clusters (several in number) in the vicinity of the kinetosomes of cirri and dorsal bristles (Fig. 8e,f,h) and occasionally in the cytoplasm away from the cortex as well (Fig. 9g–i). In particular, dozens of the granules assembled in a large vesicle in the front area of the buccal field near the endoral membrane (Figs. 7d, 9a), and occasionally near the paroral membrane (Fig. 7e). The inclusion of cortical granules could be divided into a small anterior part and a large body part; these two parts were more obvious when cortical granules located near the buccal area attaching to the pellicle (Fig. 7d,e). The small anterior part near the pellicle is often of high electron-dense while the main part is always of less electron-dense (Fig. 7d,e).

A lot of aggregations of single membrane bounded flattened saccules were found attached to the pellicle in the buccal area behind the cortical granules assemblage (Fig. 9a,b,e,f); such flattened saccules also existed in the cytoplasm near and away from the buccal area (Fig. 9d,g,h). Those near the pellicle of the buccal area were usually elongated and parallel to each other (Fig. 9a,b,e,f), while others in deeper cytoplasm were shorter or curled and sometimes encased in cytoplasmic vesicles with cortical granules and/or mitochondria inside (Fig. 9d,g,h). Cytoplasm was also rich of widespread mitochondria, cytoplasmic vesicles of varied sized, and food vacuoles (Fig. 9c,i). Endoplasmic reticulum was found occasionally and closed to mitochondria (Fig. 9l). Macronucleus was composed of a discontinuous nuclear envelope, numerous irregular shaped chromatin bodies, and nucleoli (Fig. 9j). The nuclear pores embedded in the envelope could be clearly observed (Fig. 9k).

### Molecular data and phylogenetic analyses

**Figure 10:**
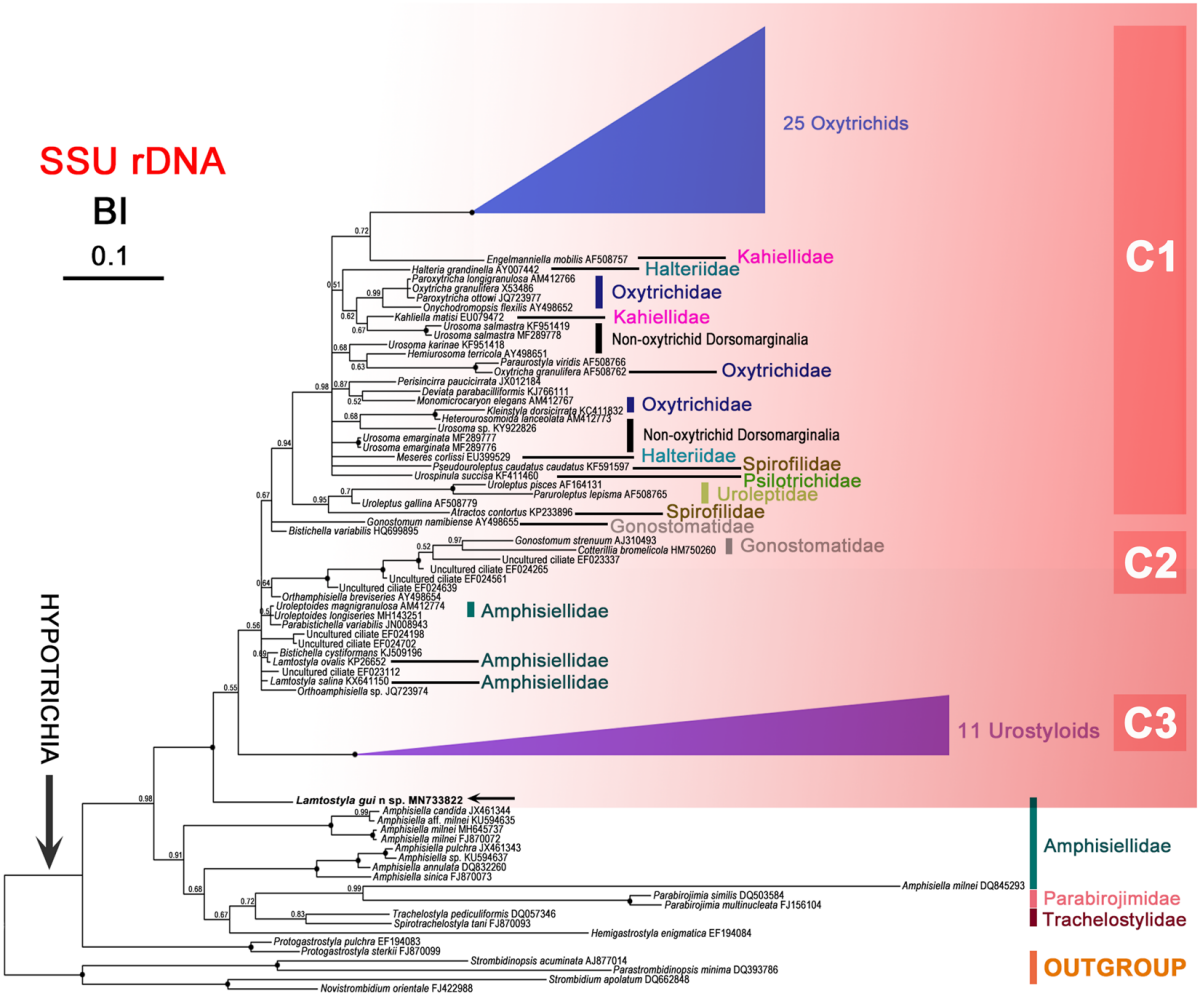
Bayesian inference (BI) analysis of small subunit (SSU) rRNA sequences data, showing the systematic position of *Lamtostyla gui* n. sp. (in bold, arrowed), based on 104 taxa and 272 sites (derived from the alignment filter 5%–98%) including gaps. Ciliate species, *Novistrombidium orientale*, *Parastrombidinopsis minima*, *Strombidinopsis acuminata* and *Strombidium apolatum* are selected as outgroup taxa. Sequences of 25 oxytrichids and 11 urostyloids are collapsed for better tree visualization (see Material and Methods for their accession numbers). Black dots indicate the full support nodes in BI analysis. All branches are drawn to the scale bar, which corresponds to ten substitution per 100 nucleotide positions. The discussed unresolved region is highlighted in red; within this region, three highly supported subclades of organisms are labelled as C1, C2 and C3.

(Fig. 10) The full ribosomal operon of *Lamtostyla gui* n. sp. was 5,238 bp long excluding PCR primers. It comprised the almost complete SSU rRNA (1,777 bp), ITS1 (131 bp), 5.8S (153 bp), ITS2 (224 bp), and almost complete LSU rRNA (2,953 bp) sequences. Overall GC content was 48.42%. The full ribosomal operon was deposited in GenBank with accession number MN733822.

In all reconstructed SSU rRNA phylogenetic trees, several poorly resolved regions could be observed. Using different alignment filters or treeing methods (i.e., maximum likelihood (ML) or bayesian inference (BI)) did not allow us to properly resolve them. In Fig. 10, one of the obtained BI trees constructed by a final 272 character matrix derived from the alignment filter 5%–98%, where columns with a similarity lower than 5% and higher than or equal to 98% had been removed and left only those informative columns, was reported as example. Although, *L. gui* apparently branched alone in the reported tree, it was indeed belonging to part of a large unresolved region with low posterior probability support. All species or subclades constituted the unresolved region were highlighted in red in Fig. 10. Within this region, three highly supported subclades of organisms emerged and were labelled as C1, C2, and C3. The reciprocal position of the three *Lamtostyla* species was not solved as each of them branched independently from others within the mentioned unresolved region. Species and subclades emerging from this unresolved region presented different evolutionary rates as evidenced by the significant differences in the length of their terminal branches. For example, *L. ovalis, L. salina*, and *Uroleptoides longiseries* (Foissner, Agatha & Berger, 2002) Berger, 2008 were showing shorter branches indicative of slower evolutionary rates; whereas, *L. gui, Cotterillia bromelicola* Foissner & Stoeck, 2011, and *Gonostomum strenuum* (Engelmann, 1862) Sterki, 1878 were showing longer branches indicative of faster evolutionary rates.

Although we characterized the almost full ribosomal operon, only the SSU rRNA gene could be used for genus/family level phylogenetic reconstruction due to the lack or extreme limitations of ITS and LSU rRNA gene sequences of related organisms. But, the ITS and LSU rRNA gene sequences of our organism represented the first available data for the genus *Lamtostyla* and one of the more complete data after *Sterkiella histriomuscorum* Foissner *et al*., 1991, GenBank accession no. FJ545743^46^.

A critical analysis of the ITS region revealed that a large number of sequences in GenBank, which shared up to 85% (usually above 90%) identity with our organism, had been annotated as uncultured fungi. With a similar level of identity, the available sequences of hypotrichs could also be retrieved. Using BLAST taxonomy reports, out of the best 1,092 hits, 748 were annotated as the phylum Ciliophora, 152 as fungi, 185 as unknown eukaryotes, 6 as unknown organisms, and 1 as moss. The information of those 152 fungal annotated hits can be found in Table S1.

## Discussion

### Comparison with closely related species

(Table 4) In the latest review of the family Amphisiellidae, Berger^3^ revised 12 species in the genus *Lamtostyla* and further separated them into three groups based on the number of cirri in ACR, the number of dorsal kineties and the presence/absence of cortical granules, i.e., (1) *Lamtostyla lamottei*-group which comprises most *Lamtostyla* species, having an ACR shorter than 50% of body length, but composed of more than four cirri, and very likely lacking cortical granules; (2) *Lamtostyla granulifera*-group, where species are characterized by having four cirri in the ACR, three dorsal kineties, and cortical granules, including *L. decorata* and *L*. *granulifera*; and (3) *Lamtostyla longa*-group, where species have basically the same cirral pattern as those in the *Lamtostyla granulifera*-group, but have five (rather than three) dorsal kineties, including *L. longa* and *L. raptans*. After that, only two species, *L*. *ovalis* and *L*. *salina*, were described, and both of them should be assigned to the *Lamtostyla lamottei*-group since they have more than four cirri (8–16 cirri in the former, 5–13 cirri in the latter) in the ACR^17,45^. Morphologically, *L. gui* n. sp. should be assigned to the *Lamtostyla granulifera*-group by having the ACR composed of four cirri, three dorsal kineties and cortical granules^3^. Hence, in total, 15 *Lamtostyla* spp. are listed below, belonging to three subgroups (Table 4).

**Table 4:** Comparison of three subgroups within the genus *Lamtostyla*, with all *Lamtostyla* species included.

Characters	*Lamtostyla lamottei*-group	*Lamtostyla granulifera*-group	*Lamtostyla longa*-group
No. of cirri in ACR	More than 4	4	4
Cortical granules	Usually lacking	Present	Not known
No. of dorsal kineties	2–4, usually 3	3	5
Species list	*L. australis*	*L. decorata*	*L. longa*
	*L. elegans*	*L. granulifera*	*L. raptans*
	*L. islandica*	*L. gui*	
	*L. lamottei*		
	*L. ovalis*		
	*L. perisincirra*		
	*L. procera*		
	*L. quadrinucleata*		
	*L. salina*		
	*L. vitiphila*		
Data sources	3, 17, 45	3, Present work	3

*ACR* amphisiellid median cirral row, *No*. number.

The other two species in the *Lamtostyla granulifera*-group, namely, *L. decorata* and *L*. *granulifera*, should be compared with our organism. Compared with *L. decorata*, *L. gui* has more (4–8, on average 5 vs. 2–4, on average 2.3) macronuclear nodules, about ten cortical granules distributed as a rosette (vs. dozens of cortical granules formed a conspicuous of plaque) around dorsal bristle, fewer pretransverse and transvers cirri (4–6 vs. 6–9), and a relatively plump body (the ratio of body length to width 3–5:1 vs. 4–9:1 *in vivo*)^47^. *Lamtostyla gui* differs from *L*. *granulifera* mainly in having (1) more (4–8, on average 5 vs. 2) macronuclear nodules; (2) smaller (0.3 μm across vs. 1–2 μm) cortical granules; and (3) fewer (28–44, on average 37 vs. 42–52, on average 45) left marginal cirri^48^.

### Morphogenetic comparison with related taxa

The most important events in the morphogenetic processes of *Lamtostyla gui* n. sp. can be summarized as follows: (1) in the proter, the posterior part of parental adoral zone of membranelles is renewed *in situ*; in the opisthe, the oral primordium is formed parakinetally from the leftmost one or two transverse cirri; (2) two sets of six FVT anlagen are formed in the middle stage; (3) four cirri in ACR originate from anlage V (posterior part) and VI (anterior part); (4) the FVT anlagen III to VI (sometimes IV to VI) provide the rearmost cirri to form the transverse cirri, while anlage II does not form a transverse cirrus at all; (5) marginal row primordia and dorsal kinety anlagen develop intrakinetally; and (6) the macronuclear nodules fuse to form a single mass before dividing. The ontogenetic processes of *L. gui* confirm it to be an amphisiellid, since it shares the main apomorphy, namely the ACR comprises cirri originated from at least two anlagen^3,49^. Moreover, detailed morphogenetic processes have previously only been reported for *L. australis* and *L. salina*, both are representatives of the *Lamtostyla lamottei*-group^3,45^; while our study on *L. gui* provides the first ontogenetic data for the *Lamtostyla granulifera*-group. A comparison of these two subgroups shows that there is only one difference between them, i.e., the parental adoral membranelles retain intact in the *Lamtostyla lamottei*-group (vs. renew partially *in situ* in the *Lamtostyla granulifera-*group)^3,45,50^. Nevertheless, since morphogenetic information are limited to few species, whether such morphogenesis difference stably supports the division of these two *Lamtostyla* subgroups or not still needs further confirmation. And the relationship of the three subgroups, or to be more precise, the inner-relationship of the genus *Lamtostyla* has not been resolved yet.

### Ultrastructure

No study about the ultrastructure of amphisiellids was previously available. Present study provides some first fine structure information for this hypotrichous group in terms of pellicle, cortical granules, ciliature, nuclear apparatus, and cytoplasm.

The pellicle is the “boundary” of the ciliates cortex, and it is sometimes covered by an additional membrane, the perilemma, and usually apposed to a layer of subpellicular microtubules^43^. Perilemma is lacking in *Lamtostyla gui*, which recalls the uncertainty of its presence in hypotrichs, i. e., it has been reported in some species of hypotrich ciliates^51–54^, whereas absent in others^55–57^. Berger^1^ discussed the correlation relationship between the arrangement of subpellicular microtubules and body rigidity in hypotrichs, that is, species with a flexible body (such as *Oxytricha fallax* Stein, 1859) bear a single layer of subpellicular microtubules, whereas species with a rigid body (such as *Stylonychia mytilus* Ehrenberg, 1838) bear subpellicular microtubules arranged in crosswise layers^54,58–60^. Such deduction is supported by present study, as *L. gui* has a soft body and meanwhile a single layer of subpellicular microtubules.

Hypotrichs share the existence of the anterior and posterior microtubular bundles originating from the rampart of marginal cirri. In several species belonging to the family Oxytrichidae Ehrenberg, 1830, small subectoplasmic rootlets to the left side of a cirrus is usually present^59,61,62^, while the linear microtubule arrays bordering the rampart wall instead the presence of small subectoplasmic rootlets may unify urostyloids^57,63,64^. In *L. gui*, the small subectoplasmic rootlets are not found, while serval microtubule bundles left of cirrus are observed, which indicates it shares more similarity with urostyloids than other hypotrichs, e.g., oxytrichids. However, in urostyloids, the linear microtubule arrays are present in both longer sides of cirri^57,63,64^, while they present only in the left side in *L. gui*. Whether the fine structure of associated microtubules reflecting systematic position or not may deserve further discussion.

Pharyngeal discs existed in the oral cortex have been reported in many ciliate taxa and considered as food vacuole membrane precursors^65–69^. For ciliates in the subclass Hypotrichia, limited reports regarding the morphology of pharyngeal discs are available, i.e., only in the genus *Stylonychia* Ehrenberg, 1830 and *Thigmokeronopsis* Wicklow, 1981, where there are small vesicles-like structures distributed in the cytoplasm of oral area^59,70^. The flattened saccules existed in the buccal area of *L. gui* are pharyngeal discs considering their location and membrane nature. However, they represent a different form from those in *Stylonychia* and *Thigmokeronopsis*. This might be related with the systematic assignment of these three species, i.e., *Stylonychia* and *Thigmokeronopsis* belong to the family Oxytrichidae and Pseudokeronopsidae Borror & Wicklow, 1983, respectively, whereas, *Lamtostyla* represents the family Amphisiellidae^1–3^.

Cortical granules widely distribute in the cortex of soft hypotrichous ciliates and some of them are confirmed to be extrusomes^1–4,8,71–75^. Hitherto, four types of extrusomes: trichocysts-like extrusomes (e.g., in *Pseudourostyla cristata* (Jerka-Dziadosz, 1964) Borror, 1972), mucocysts (e.g., in *Urostyla grandis* Ehrenberg, 1830), pigmentocysts (e.g., in *Pseudokeronopsis carnea* Cohn, 1866), and cup-shaped extrusomes (e.g., in *Oxytricha granulifera* Foissner & Adam, 1983) have been documented for hypotrichs^57,73–76^. The cortical granules in *Lamtostyla gui* are membrane-bounded structures, thus, are potential extrusomes^77^. Considering their morphological characters, i.e., ellipsoidal in shape with bipartite inclusion, they might be assigned to mucocysts, however, considering their distribution, i. e., mainly around bases of cirri and dorsal bristles, they are quiet similar with cortical ampules in the subclass Euplotia Jankowski, 1979^78,79^. Moreover, they are largely packed forehead of the pharyngeal discs in the buccal area, hence, we assume that they might be helpful in food ingestion or pretreatment. Further studies are needed to test their ability of extrusion and their functions.

### Marker gene sequences and phylogenetic analyses

In the present study, we characterized the almost complete ribosomal operon of *Lamtostyla* *gui* n. sp. The available SSU rRNA data only allows us to observe that *L. ovalis* and *L. salina* (both belonging to the *Lamtostyla lamottei*-group) show an identity of 99.58% that is higher than 98.21% and 97.98% which is the identity they respectively present with *L. gui* (belonging to the *Lamtostyla granulifera*-group). Additionally, *L. gui* presents an apparently higher evolutionary rate compared with other two species in the phylogenetic tree. The present SSU rRNA phylogenetic tree does not support nor deny the possible monophyly of the genus *Lamtostyla*, which is in agreement with previous studies^17,45^ and the phylogenetic position of *Lamtostyla* still remains undetermined, since the present topology is rather unstable with low support values across the tree and the lack of molecular phylogenetic information for the type species. Moreover, our species also shows high similarity in the SSU rRNA gene sequences of some familial unknown hypotrichs, namely, *Bistichella cystiformans* KJ509196 (98.03%), *Bistichella variabilis* HQ699895 (98.10%), *Orthoamphisiella breviseries* AY498654 (97.99%), *Orthoamphisiella* sp. JQ723974 (98.03%), *Parabistichella variabilis* JN008943 (98.15%), *Uroleptoides longiseries* MH143251 (97.68%) and *Uroleptoides magnigranulosa* AM412774 (98.27%). It is worth to reminding that the reciprocal positions of all these species or clades are rather unstable; indeed they emerge from the region which is highlighted in red (Fig. 10) where relative positions are not properly resolved using only SSU rRNA gene sequences (posterior probability values lower than 0.50). Consequently, any discussion about molecular similarity among these organisms/clades is, at present, only speculative and other molecular markers will be necessary in the future to properly resolve the issue. It is intriguing that, morphologically, *Lamtostyla* significantly differs from *Bistichella* Berger, 2008 in usually having one buccal cirrus (vs. more than one) and a short ACR originated from anlage V and anlage VI (vs. two separately long frontoventral cirral rows originated from V and VI, which are much longer than the ACR)^3,9,80^. The genus *Orthoamphisiella* Eigner & Foissner, 1991 differs from *Lamtostyla* in having a long frontoventral cirral row forming from a single anlage (vs. from at least two anlagen), more than one buccal cirrus (vs. usually one), and transverse cirri absent (vs. present)^3,4^. The genus *Parabistichella* Jiang *et al*., 2013 is a bakuellid-like hypotrichs with a midventral complex composed of pairs and row which is absent in *Lamtostyla*; beside, over six frontoventral-transverse cirri anlagen are present during the division of *Parabistichella*, whereas *Lamtostyla* usually have six frontoventral-transverse cirri anlagen^3,13^. The type species in both *Lamtostyla* and *Uroleptoides* Wenzel, 1953 are not well described morphologically and molecularly, causing the uncertain familial classifications of those two genera; now, they are preliminary recognized as amphisiellids with only one pragmatic solution to separate them, that is, by the length of ACR (more than 50% of body length in *Uroleptoides* against less than 50% in *Lamtostyla*)^3^.

In order to verify the monophyly of *Lamtostyla* as well as many other genera of hypotrichs, additional molecular markers showing a higher evolutionary rate should be used, such as ITS or mitochondrial cytochrome oxidase subunit 1 (COI) gene. Indeed, in the genera where SSU rRNA presents similar features, like *Tetrahymena* Furgason, 1940 and *Paramecium* Müller, 1773, these additional markers are routinely characterized and used for fast identification^81–86^. Additionally, the combined phylogenetic analyses of multiple genes (e. g., SSU rRNA, ITS1-5.8S-ITS2 and LSU rRNA or phylogenomic data) with increased sampling are now becoming popular within ciliates^87–91^, also in the Hypotrichia^31,32,34,35,38^, to study the evolutionary relationships, and are revealing to provide more robust interpretations. Hence, it becomes reasonable that as many as possible molecular marker genes of hypotrichous species should be characterized and added to species description or re-description to provide more information in solving complex systematics problems.

Concerning ITS and LSU rRNA data, due to the present lack of sequences from related species, we could not use these data for phylogenetic analyses, and especially, to test the monophyly of *Lamtostyla*. As an additional note, it is worth mentioning that the properly taxonomically annotated complete ribosomal operon sequence we produced, in particular the ITS region, will help correcting mis-annotations present in GenBank. ITS region is commonly used as the phylogenetic marker to identify fungi in culture independent studies^92–95^. The performed GenBank survey using ITS1-5.8S-ITS2 sequencing data of *L. gui*, highlights the presence of up to 152 sequences attributed to fungi, which are likely misannotated deriving either from the phylum Ciliophora or being of the chimeric origin. In addition to reveal probable mis-annotations, our survey also highlights the presence of ciliates, especially hypotrichs, in the environment/matrices generally studied for the presence of fungi. Intriguingly, most of these environment/matrices represent human byproducts, e.g., compost^96^, forest soil^97,98^, or even extreme environment, e. g., crater lake^99^ and permafrost^100^, which should deserve further investigations from the community of ciliate taxonomists.

### Taxonomic summary

Order Stichotrichida Fauré-Fremiet, 1961

Family Amphisiellidae Jankowski, 1979

Genus *Lamtostyla* Buitkamp, 1977

*Lamtostyla gui* n. sp.

**Diagnosis** Size *in vivo* 134–183 × 30–49 μm. Body very flexible, slenderly elliptical and often slightly sigmoidal. Cortical granules small and colorless, inconspicuously scattered around the marginal cirrus and dorsal bristle. 20–30 adoral membranelles. Amphisiellid median cirral row composed of four or five cirri, with three to five frontoventral cirri on its left. Invariable three frontal cirri and one buccal cirrus. Usually four transverse cirri and one pretransverse cirrus. One left and one right marginal row, composed of 28–44 and 30–45 cirri respectively. Usually four ellipsoidal to binodal macronuclei and three micronuclei. Three dorsal kineties.

**Type locality** Marsh wetland (31°36′50.22″N, 121°49′5.27″E, Chongming Island, Shanghai, China) covered with *Spartina alterniflora* Loisel as dominant vegetation. Water temperature was 12 °C and salinity was approximately 10‰. For more details, see Material and Methods.

**Type specimens** Two protargol-impregnated slides, one containing holotype specimen (Figs. 1b,c, 2h,i; registry number: LWY2015111409-1) and the other containing several paratypes (registry number: LWY2015111409-2) marked by black circle have been deposited in the Laboratory of Protozoology, East China Normal University, China.

**Dedication** We dedicate this species to Prof. Fukang Gu, School of Life Sciences, East China Normal University, for his dedication to the cytology of protozoan and also as a small token of appreciation for his kind guide and support for Ms. Wanying Liao during her master study.

**ZooBank registration number of present work** urn:lsid:zoobank.org:pub:FB685150-BFF5-478F-9880-7D2C2EF773BC.

**ZooBank registration number of genus** ***Lamtostyla*** **Buitkamp, 1977** urn:lsid:zoobank.org:act:962F7B2A-7CFA-47AF-8A78-EAAF33249041.

**ZooBank registration number of** ***Lamtostyla gui*** **n. sp**. urn:lsid:zoobank.org:act:CAFA9428-BD21-4FCE-94E3-49A9C5A15EA1.

## Material and Methods

### Sample collection, cultivation and identification

The mixtures of water and sediments were collected with clean jars on 14 November, 2015 from the surface of a marsh wetland in North Beach (31°36′50.22″N, 121°49′5.27″E), Chongming Island, Shanghai, China. The water temperature was 12 °C, salinity was 10‰ and pH was 8.2. Samples were transported to laboratory, then, poured out in Petri dishes and maintained at room temperature (about 24 °C). Pre-sterilized wheat grains were added to the Petri dishes to enrich the growth of bacterial food for ciliates. *Lamtostyla gui* n. sp. appeared one week later in the raw culture, together with *Urosoma salmastra* (Dragesco & Dragesco-Kernéis, 1986) Berger, 1999. The feeding of *L. gui* on *U. salmastra* caused the acute decline of the latter and consequently the decline of itself within a short period. Later, *L. gui* throve again with the explosion of an unidentified scuticociliate. Two uniprotistan cultures were set up, each starting with about ten isolated cells from the raw sampling. In one culture, a wheat grain was added to enrich bacterial food, whereas, in the other, *L. gui* was feed with pure culture of the unidentified scuticociliate.

Living cells were directly isolated from the Petri dishes using micropipettes and observed with bright field and differential interference contrast microscope (Olympus BX 51) at a magnification of 100–1,000×. Commercial protargol powder was used following staining protocol^101^ to reveal the nuclear apparatus and the infraciliature. Stained specimens were counted and measured at a magnification of 400–1,000×, while drawings were made with the aid of a camera lucida at a magnification of 1,250×. In illustrations of morphogenetic processes, old (parental) ciliary structures were depicted by contours whereas new structures were shaded black. Systematics and terminology are mainly according to Berger' s monograph^3^.

### Electron microscopy

For SEM, the specimens from one poly-clonal culture using bacteria as food source were fixed in a 1:6 mixture with 1% O_S_O_4_ in 0.1 M cacodylate buffer (pH 7.2) and saturated solution of HgCl_2_ in distilled water for 10 mins at room temperature; then cells were washed three times in 0.1 M phosphate buffer (pH 7.0) to remove fixation solution; after alcohol dehydrations and critical point drying by CO_2_, cells were finally coated with platinum; observation was conducted under a Hitachi S-4800 scanning electron microscope with accelerating voltage of 5.0 kV.

Transmission electron microscopy (TEM) preparation was obtained by fixing specimens from one poly-clonal culture using bacteria as food source in a 1:1 mixture of 2% OsO_4_ in 0.2 M phosphate buffer (pH 7.0) and 2.5% glutaraldehyde in 0.2 M cacodylate buffer (pH 7.2) for 10 mins at 4 °C; then after three times washes in 0.2 M cacodylate buffer, cells were post-fixed in 1% phosphate buffered OsO_4_ for 1 h at 4 °C; after three times washes in buffer again, specimens were undertaken alcohol dehydrations, acetone dehydrations, embedded in Epon 12, and then polymerized at 37 °C for 16 h, 45 °C for 24 h and 60 °C for 48 h. Thin sections were cut with a diamond knife and then placed on copper grids using uranyl acetate and lead citrate for staining; finally, the sections were observed under a Hitachi HT7700 transmission electron microscope with accelerating voltage of 100 kV.

### DNA extraction, polymerase chain reaction (PCR) amplification and sequencing

Two cells form one poly-clonal culture using bacterial food source were washed three times in distilled water and then conducted total genomic DNA extraction using the DNeasy Tissue Kit (Qiagen, Hilden, Germany), following the manufacture’s instruction. The SSU rRNA gene sequence was amplified by touchdown PCR, using Q5^®^ Hot Start High-Fidelity DNA Polymerase (New England BioLabs version cat. no. M0494S) and the universal primers^102^. Cycling parameters for PCR amplifications were as follows: 98 °C for 30 s, followed by 17 cycles of 98 °C for 10 s, 67 °C (decreasing by 1 °C per cycle) for 30 s, and 72 °C for 1 min; and followed by 18 cycles of 98 °C for 10 s, 50 °C for 30 s, and 72 °C for 1 min; then 72 °C for 5 mins for final extension. Cloning was performed using the *pEASY*^®^-Blunt Cloning Kit (TransGen Biotech, Beijing, China) following the manufacture’s instruction. Bidirectional sequencing was performed by the Thermo Fisher Scientific China Co. Ltd. (Shanghai, China) using the M13-47 and M13-48 primers. Furthermore, in order to avoid mismatches in the sequencing results obtained from cloning product, direct sequencings of a new purified PCR product (NucleoSpin^®^ Extract II Kit, Macherey-Nagel, Germany) were also made (GATC Biotech AG, European Custom Sequencing Centre, Germany) using the internal primers R536, F783, and R1052^103^. Results were 100% identical.

The ITS and LSU rRNA gene sequences were amplified with the forward primer matching a sequence on the SSU rRNA gene and the reverse primer matching a sequence on the LSU rRNA gene (FG1400 5′-TTGYACACACCGCCCGTC-3′^81^ and 28S R2992 5′- AAACTAACCTGTCTCACGACGGTC-3′, respectively) under the following conditions: 3 mins initial denaturation (94 °C); 35 cycles of 30 s at 94 °C, 30 s at 55 °C and 150 s at 72 °C and a final extension step of 6 mins (72 °C), together with high-fidelity Takara Ex Taq PCR reagents (Takara Bio Inc., Otsu, Japan) according to the manufacture’s instruction. Then, purified PCR products were sent for direct sequencing in both directions by using the PCR forward primers, FG1400, and some appropriate internal primers: RGD2 5′-GGTCCGTGTTTCAAGACGGG-3′^81^, 28S F1993 5′-TTGGGGGATTGGCTCTGAGG-3′, 28S R1318 5′-TCGGCAGGTGAGTTGTTACACAC-3′, and 28S R2219 5′-CAGAGCACTGGGCAGAAATCAC-3′.

### Phylogenetic analyses based on SSU rRNA

The obtained SSU rRNA sequences were assembled using Chromas Lite 2.1 software and compared with the non-redundant sequence database using NCBI-BLAST^104^, then aligned using the editor and alignment tools from the ARB program package^105^ together with related sequences contained in the SSU rRNA SILVA 102 database^106,107^ and some latest released amphisiellids sequences on GenBank database. The alignment was then corrected taking into account the base-pairing scheme in rRNA secondary structure. Similarity values among sequences were calculated using the appropriate tool from the ARB software package, after trimming the PCR primer region. Phylogenetic analyses were performed on a selection of 104 SSU rRNA gene sequences of the subclasses Hypotrichia, Choreotrichia Small & Lynn, 1985 and Oligotrichia Bütschli, 1887, including the new sequence of our species. Among them, *Novistrombidium orientale* Liu *et al*., 2009, *Parastrombidinopsis minima* Tsai *et al*., 2008, *Strombidinopsis acuminata* Fauré-Fremiet, 1924, and *Strombidium apolatum* Wilbert & Song, 2005 were selected as outgroup taxa. The GenBank accession numbers of 25 oxytrichids and 11 urostyloids are as follows: *Cyrtohymena citrina* AF164135, *Cyrtohymena muscorum* KM061384, *Gastrostyla steinii* AF508758, *Hemiurosomoida longa* AF164125, *Histriculus histrio* FM209294, *Laurentiella strenua* AJ310487, *Neokeronopsis aurea* EU124669, *Onychodromus grandis* AJ310486, *Onychodromus quadricornutus* X53485, *Paraurosomoida indiensis* JX139117, *Paraurostyla weissei* AF164127, *Pattersoniella vitiphila* AJ310495, *Pleurotricha lanceolata* AF508768, *Pseudogastrostyla flava* KP266627, *Rigidohymena candens* KC414885, *Rubrioxytricha ferruginea* AF370027, *Steinia sphagnicola* AJ310494, *Sterkiella histriomuscorum* AF508770, *Stylonychia ammermanni* FM209295, *Stylonychia bifaria* FM209296, *Stylonychia lemnae* AF164124, *Stylonychia mytilus* AF164123, *Stylonychia notophora* FM209297, *Tetmemena pustulata* AF508775, *Urosomoida agilis* KJ864926; *Anteholosticha manca* DQ503578, *Diaxonella trimarginata* DQ190950, *Metaurostylopsis salina* EU220229, *Metaurostylopsis struederkypkeae* EU220228, *Nothoholosticha fasciola* FJ377548, *Pseudokeronopsis carnae* AY881633, *Pseudokeronopsis erythrina* FJ775723, *Thigmokeronopsis stoecki* EU220226, *Uroleptopsis citrina* FJ870094, *Uroleptopsis citrina* GU437211, *Urostyla grandis* AF164129. The accession umbers of other sequences were provided in Fig. 10. Out of the species selection and alignment, we produced different databases for later analysis. These databases were produced applying filters to retain or exclude columns whose overall similarity was either, 5% or below, or 98% or above (gap was counted as a fifth character for similarity calculation). The rationale for excluding low similarity region (5% or below) was to reduce possible “noise” (saturated signal) from hypervariable region. The rationale to exclude columns with a similarity of 98% or higher was to exclude columns containing identical characters (100% identity, uninformative) and columns in which only one sequence out of 104 was different from the others (99% identity). Indeed, in the last case, the single difference in the highly conserved region could be the result of a sequencing error. ML trees were calculated with the PHYML software version 2.4^108^ from the ARB package, performing 100 pseudo-replicates. BI analyses were performed with MrBayes 3.2^109^ using three runs each with one cold and three heated Monte Carlo Markov chains, with a burn-in of 25%, iterating for 1,000,000 generations. Analyses were performed with the different generated filters and topologies of the trees compared.

## Supplementary information

Table S1.
