# Supplementary material for: Documentation of a new hypotrich species in the family Amphisiellidae, Lamtostyla gui n. sp. (Protista, Ciliophora) using a multidisciplinary approach
Source: Sci Rep. 2020 Feb 28;10:3763. doi: 10.1038/s41598-020-60327-5 (PMC7048753; doi:10.1038/s41598-020-60327-5)
Supplement: Supplementary file 1 — Table S1. [file 41598_2020_60327_MOESM1_ESM.docx]

SCIENTIFIC REPORTS

**Supplementary information**

**Documentation of a new hypotrich species in the family Amphisiellidae, *Lamtostyla gui* n. sp. (Protista, Ciliophora) using a multidisciplinary approach**

**Wanying Liao^1, 2^, Zhiwei Gong^3^, Bing Ni^1^, Xinpeng Fan1 & Giulio Petroni^2^**

1 School of Life Sciences, East China Normal University, Shanghai 200241, China

2 Department of Biology, University of Pisa, Via Alessandro Volta 4, Pisa 56126, Italy

3 School of Physics and Electronic Science, East China Normal University, Shanghai 200241, China

Correspondence and requests for materials should be addressed to X. F. (email: [xpfan@bio.ecnu.edu.cn](mailto:xpfan@bio.ecnu.edu.cn))

**Table S1. List of 152 fungal annotated hits in the BLAST taxonomy reports derived from the analysis of internal transcribed spacers (ITS) region of *Lamtostyla gui* n. sp.**

| No. | GenBank Acc. No. | Taxon label | Length | Isolation source | Country, Place | Data origins |
| --- | --- | --- | --- | --- | --- | --- |
| 1 | KT581718 | Uncultured fungus clone WPW-OTU-16 | 572 bp | Quercus deserticola litter | Mexico | Published paper^[1]^ |
| 2 | KT581669 | Uncultured fungus clone WPD-OTU-27 | 583 bp | Quercus deserticola litter | Mexico | Published paper^[1]^ |
| 3 | KC884302 | Uncultured fungus clone 4_117 | 583 bp | Permafrost soil samples | China, Qinghai-Tibet Plateau | Published paper^[2]^ |
| 4 | KC884311 | Uncultured fungus clone 4_362 | 583 bp | Permafrost soil samples | China, Qinghai-Tibet Plateau | Published paper^[2]^ |
| 5 | DQ900960 | Uncultured fungus clone gbCTA5_003 | 820 bp | Composting woodchips and yard debris | USA, City of Durham | Published paper^[3]^ |
| 6 | DQ900996 | Uncultured fungus clone gbFC_3b6_011 | 846 bp | Composting woodchips and yard debris | USA, City of Durham | Published paper^[3]^ |
| 7 | DQ900965 | Uncultured fungus clone gbCTA18_025 | 800 bp | composting woodchips and yard debris | USA, City of Durham | Published paper^[3]^ |
| 8 | DQ900977 | Uncultured fungus clone gbCTA54_059 | 854 bp | composting woodchips and yard debris | USA, City of Durham | Published paper^[3]^ |
| 9 | DQ900971 | Uncultured fungus clone gbCTA35_034 | 748 bp | composting woodchips and yard debris | USA, City of Durham | Published paper^[3]^ |
| 10 | DQ900966 | Uncultured fungus clone gbCTA29_023 | 532 bp | composting woodchips and yard debris | USA, City of Durham | Published paper^[3]^ |
| 11 | DQ900967 | Uncultured fungus clone gbCTA30_031 | 364 bp | composting woodchips and yard debris | USA, City of Durham | Published paper^[3]^ |
| 12 | AY704760 | Uncultured soil fungus isolate OTU-ZY30 | 377 bp | Agricultural soil | / | Published paper^[4]^ |
| 13 | AY704749 | Uncultured soil fungus isolate OTU-ZY19 | 378 bp | Agricultural soil | / | Published paper^[4]^ |
| 14 | KP897235 | Uncultured fungus clone 2168_72 | 349 bp | *Picea abies* needles | Lithuania | Published paper^[5]^ |
| 15 | KP897591 | Uncultured fungus clone 2168_481 | 360 bp | *Picea abies* needles | Lithuania | Published paper^[5]^ |
| 16 | EU709516 | Uncultured fungus isolate DGGE gel band 4 | 358 bp | Cellulose biofilm in stream | USA, Michigan | Published paper^[6]^ |
| 17 | EU709515 | Uncultured fungus isolate DGGE gel band | 357 bp | Cellulose biofilm in stream | USA, Michigan | Published paper^[6]^ |
| 18 | MG132663 | Uncultured fungus clone pollen442 | 676 bp | Crater Lake pollen | / | Published paper^[7]^ |
| 19 | MG132654 | Uncultured fungus clone Pollen427 | 640 bp | Crater Lake pollen | / | Published paper^[7]^ |
| 20 | MG132653 | Uncultured fungus clone Pollen426 | 527 bp | Crater Lake pollen | / | Published paper^[7]^ |
| 21 | MG132643 | Uncultured fungus clone pollen411 | 781 bp | Crater Lake pollen | / | Published paper^[7]^ |
| 22 | JQ312937 | Uncultured fungus clone 188_GKG40MV01CJ4EJ | 337 bp | *Picea abies* forest soil | / | Published paper^[8]^ |
| 23 | JQ312859 | Uncultured fungus clone 141_GKG40MV01AO1SV | 344 bp | *Picea abies* forest soil | / | Published paper^[8]^ |
| 24 | KM493018 | Uncultured fungus clone 454_73 | 289 bp | Norway spruce decaying log | Sweden | Published paper^[9]^ |
| 25 | KM493254 | Uncultured fungus clone 454_294 | 289 bp | Norway spruce decaying log | Sweden | Published paper^[9]^ |
| 26 | KM493026 | Uncultured fungus clone 454_81 | 290 bp | Norway spruce decaying log | Sweden | Published paper^[9]^ |
| 27 | FJ553511 | Uncultured Agaricomycetes clone LTSP_EUKA_P3J23 | 877 bp | Forest soil | Canada, Skulow Lake | Published paper^[10]^ |
| 28 | JX974769 | Uncultured fungus clone B2103 | 948 bp | Polluted estuarine sediment | China, Laizhou Bay | Unpublished paper^[a]^ |
| 29 | JX974770 | Uncultured fungus clone D232 | 936 bp | Polluted estuarine sediment | China, Laizhou Bay | Unpublished paper^[a]^ |
| 30 | MF971347 | Uncultured fungus clone OTU322 | 619 bp | Soil | China, Tibet Plateau | Unpublished paper^[b]^ |
| 31 | MF971519 | Uncultured fungus clone OTU553 | 621 bp | Soil | China, Tibet Plateau | Unpublished paper^[b]^ |
| 32 | MF971367 | Uncultured fungus clone OTU361 | 603 bp | Soil | China, Tibet Plateau | Unpublished paper^[b]^ |
| 33 | MF971357 | Uncultured fungus clone OTU342 | 624 bp | Soil | China, Tibet Plateau | Unpublished paper^[b]^ |
| 34 | MF971362 | Uncultured fungus clone OTU351 | 591 bp | Soil | China, Tibet Plateau | Unpublished paper^[b]^ |
| 35 | MF971358 | Uncultured fungus clone OTU344 | 592 bp | Soil | China, Tibet Plateau | Unpublished paper^[b]^ |
| 36 | MF971360 | Uncultured fungus clone OTU348 | 595 bp | Soil | China, Tibet Plateau | Unpublished paper^[b]^ |
| 37 | MF971343 | Uncultured fungus clone OTU315 | 626 bp | Soil | China, Tibet Plateau | Unpublished paper^[b]^ |
| 38 | MF971344 | Uncultured fungus clone OTU317 | 600 bp | Soil | China, Tibet Plateau | Unpublished paper^[b]^ |
| 39 | MF971361 | Uncultured fungus clone OTU349 | 590 bp | Soil | China, Tibet Plateau | Unpublished paper^[b]^ |
| 40 | MF971348 | Uncultured fungus clone OTU327 | 615 bp | Soil | China, Tibet Plateau | Unpublished paper^[b]^ |
| 41 | MF971366 | Uncultured fungus clone OTU360 | 591 bp | Soil | China, Tibet Plateau | Unpublished paper^[b]^ |
| 42 | MF971350 | Uncultured fungus clone OTU329 | 599 bp | Soil | China, Tibet Plateau | Unpublished paper^[b]^ |
| 43 | MF971359 | Uncultured fungus clone OTU346 | 612 bp | Soil | China, Tibet Plateau | Unpublished paper^[b]^ |
| 44 | MF971341 | Uncultured fungus clone OTU313 | 614 bp | Soil | China, Tibet Plateau | Unpublished paper^[b]^ |
| 45 | MF971108 | Uncultured fungus clone OTU38 | 584 bp | Soil | China, Tibet Plateau | Unpublished paper^[b]^ |
| 46 | MF971342 | Uncultured fungus clone OTU314 | 582 bp | Soil | China, Tibet Plateau | Unpublished paper^[b]^ |
| 47 | MF971105 | Uncultured fungus clone OTU31 | 619 bp | Soil | China, Tibet Plateau | Unpublished paper^[b]^ |
| 48 | MF971169 | Uncultured fungus clone OTU116 | 611 bp | Soil | China, Tibet Plateau | Unpublished paper^[b]^ |
| 49 | MF971365 | Uncultured fungus clone OTU359 | 625 bp | Soil | China, Tibet Plateau | Unpublished paper^[b]^ |
| 50 | MF971107 | Uncultured fungus clone OTU37 | 595 bp | Soil | China, Tibet Plateau | Unpublished paper^[b]^ |
| 51 | MF971106 | Uncultured fungus clone OTU36 | 617 bp | Soil | China, Tibet Plateau | Unpublished paper^[b]^ |
| 52 | MF971662 | Uncultured fungus clone OTU738 | 605 bp | Soil | China, Tibet Plateau | Unpublished paper^[b]^ |
| 53 | MF971351 | Uncultured fungus clone OTU330 | 549 bp | Soil | China, Tibet Plateau | Unpublished paper^[b]^ |
| 54 | MF971661 | Uncultured fungus clone OTU736 | 625 bp | Soil | China, Tibet Plateau | Unpublished paper^[b]^ |
| 55 | MF971678 | Uncultured fungus clone OTU760 | 625 bp | Soil | China, Tibet Plateau | Unpublished paper^[b]^ |
| 56 | MF971665 | Uncultured fungus clone OTU742 | 515 bp | Soil | China, Tibet Plateau | Unpublished paper^[b]^ |
| 57 | MF971368 | Uncultured fungus clone OTU362 | 634 bp | Soil | China, Tibet Plateau | Unpublished paper^[b]^ |
| 58 | MF971675 | Uncultured fungus clone OTU752 | 613 bp | Soil | China, Tibet Plateau | Unpublished paper^[b]^ |
| 59 | MF971676 | Uncultured fungus clone OTU756 | 628 bp | Soil | China, Tibet Plateau | Unpublished paper^[b]^ |
| 60 | MF971679 | Uncultured fungus clone OTU762 | 607 bp | Soil | China, Tibet Plateau | Unpublished paper^[b]^ |
| 61 | MF971857 | Uncultured fungus clone OTU963 | 608 bp | Soil | China, Tibet Plateau | Unpublished paper^[b]^ |
| 62 | MF971677 | Uncultured fungus clone OTU757 | 584 bp | Soil | China, Tibet Plateau | Unpublished paper^[b]^ |
| 63 | MF971699 | Uncultured fungus clone OTU791 | 590 bp | Soil | China, Tibet Plateau | Unpublished paper^[b]^ |
| 64 | MF971663 | Uncultured fungus clone OTU739 | 615 bp | Soil | China, Tibet Plateau | Unpublished paper^[b]^ |
| 65 | MF971520 | Uncultured fungus clone OTU556 | 474 bp | Soil | China, Tibet Plateau | Unpublished paper^[b]^ |
| 66 | MF971968 | Uncultured fungus clone OTU1101 | 701 bp | Soil | China, Tibet Plateau | Unpublished paper^[b]^ |
| 67 | MF971967 | Uncultured fungus clone OTU1100 | 700 bp | Soil | China, Tibet Plateau | Unpublished paper^[b]^ |
| 68 | MF971168 | Uncultured fungus clone OTU115 | 513 bp | Soil | China, Tibet Plateau | Unpublished paper^[b]^ |
| 69 | MF972042 | Uncultured fungus clone OTU1183 | 658 bp | Soil | China, Tibet Plateau | Unpublished paper^[b]^ |
| 70 | MF971522 | Uncultured fungus clone OTU562 | 618 bp | Soil | China, Tibet Plateau | Unpublished paper^[b]^ |
| 71 | MF971364 | Uncultured fungus clone OTU357 | 578 bp | Soil | China, Tibet Plateau | Unpublished paper^[b]^ |
| 72 | MF971363 | Uncultured fungus clone OTU354 | 322 bp | Soil | China, Tibet Plateau | Unpublished paper^[b]^ |
| 73 | MF971521 | Uncultured fungus clone OTU561 | 595 bp | Soil | China, Tibet Plateau | Unpublished paper^[b]^ |
| 74 | HM131983 | Uncultured soil fungus clone D2I9 | 550 bp | Soil | China | Unpublished paper^[c]^ |
| 75 | HM131999 | Uncultured soil fungus clone D1I20 | 574 bp | Soil | China | Unpublished paper^[c]^ |
| 76 | KX826893 | Uncultured fungus clone TTE28 | 560 bp | Wheat seedlings | China, farm in Nanjing | Unpublished paper^[d]^ |
| 77 | KP843453 | Uncultured fungus clone W107 | 534 bp | *Magnolia soulangeana* | China, Wuhouci temple | Unpublished paper^[e]^ |
| 78 | KP843454 | Uncultured fungus clone W108 | 534 bp | *Magnolia soulangeana* | China, Wuhouci temple | Unpublished paper^[e]^ |
| 79 | KP843449 | Uncultured fungus clone W57 | 534 bp | *Magnolia soulangeana* | China, Wuhouci temple | Unpublished paper^[e]^ |
| 80 | KY978242 | Uncultured fungus clone S10 | 578 bp | Atmospheric air | India, Chennai | Unpublished paper^[f]^ |
| 81 | KY322081 | Uncultured fungus clone AO11 | 340 bp | Rhizosphere | Oman | Unpublished paper^[g]^ |
| 82 | KY322067 | Uncultured fungus clone CQ6 | 340 bp | Rhizosphere | Oman | Unpublished paper^[g]^ |
| 83 | KY322015 | Uncultured fungus clone AD35 | 340 bp | Rhizosphere | Oman | Unpublished paper^[g]^ |
| 84 | KY322063 | Uncultured fungus clone CQ2 | 340 bp | Rhizosphere | Oman | Unpublished paper^[g]^ |
| 85 | KY322061 | Uncultured fungus clone CA17 | 340 bp | Rhizosphere | Oman | Unpublished paper^[g]^ |
| 86 | KY322000 | Uncultured fungus clone AD20 | 340 bp | Rhizosphere | Oman | Unpublished paper^[g]^ |
| 87 | KY694747 | Uncultured fungus clone BS.DW2.3 | 340 bp | Rhizosphere | / | Unpublished paper^[h]^ |
| 88 | KY690136 | Uncultured fungus clone BS.DW1.11 | 340 bp | Rhizosphere | / | Unpublished paper^[h]^ |
| 89 | KX193229 | Uncultured fungus clone 808 | 340 bp | Soil | USA, Illinois | Unpublished paper^[i]^ |
| 90 | KX192915 | Uncultured fungus clone 494 | 339 bp | Soil | USA, Illinois | Unpublished paper^[i]^ |
| 91 | KX194418 | Uncultured fungus clone 1997 | 340 bp | Soil | USA, Illinois | Unpublished paper^[i]^ |
| 92 | KX192839 | Uncultured fungus clone 418 | 340 bp | Soil | USA, Illinois | Unpublished paper^[i]^ |
| 93 | KX195192 | Uncultured fungus clone 2771 | 336 bp | Soil | USA, Illinois | Unpublished paper^[i]^ |
| 94 | KX195984 | Uncultured fungus clone 3563 | 337 bp | Soil | USA, Illinois | Unpublished paper^[i]^ |
| 95 | KX194128 | Uncultured fungus clone 1707 | 338 bp | Soil | USA, Illinois | Unpublished paper^[i]^ |
| 96 | KX195507 | Uncultured fungus clone 3086 | 338 bp | Soil | USA, Illinois | Unpublished paper^[i]^ |
| 97 | KX193387 | Uncultured fungus clone 966 | 336 bp | Soil | USA, Illinois | Unpublished paper^[i]^ |
| 98 | KX194537 | Uncultured fungus clone 2116 | 339 bp | Soil | USA, Illinois | Unpublished paper^[i]^ |
| 99 | KX194141 | Uncultured fungus clone 1720 | 339 bp | Soil | USA, Illinois | Unpublished paper^[i]^ |
| 100 | KX193560 | Uncultured fungus clone 1139 | 337 bp | Soil | USA, Illinois | Unpublished paper^[i]^ |
| 101 | KX193147 | Uncultured fungus clone 726 | 339 bp | Soil | USA, Illinois | Unpublished paper^[i]^ |
| 102 | KX195035 | Uncultured fungus clone 2614 | 338 bp | Soil | USA, Illinois | Unpublished paper^[i]^ |
| 103 | KX194108 | Uncultured fungus clone 1687 | 339 bp | Soil | USA, Illinois | Unpublished paper^[i]^ |
| 104 | KX195481 | Uncultured fungus clone 3060 | 341 bp | Soil | USA, Illinois | Unpublished paper^[i]^ |
| 105 | KX194118 | Uncultured fungus clone 1697 | 328 bp | Soil | USA, Illinois | Unpublished paper^[i]^ |
| 106 | KX194962 | Uncultured fungus clone 2541 | 327 bp | Soil | USA, Illinois | Unpublished paper^[i]^ |
| 107 | MF569437 | Uncultured fungus clone 829 | 380 bp | Soil | / | Direct Submission by Beck, A. M. & Yannarell, A. C. |
| 108 | MF569932 | Uncultured fungus clone 1400 | 380 bp | Soil | / | Direct Submission by Beck, A. M. & Yannarell, A. C. |
| 109 | MF569552 | Uncultured fungus clone 964 | 379 bp | Soil | / | Direct Submission by Beck, A. M. & Yannarell, A. C. |
| 110 | MF569415 | Uncultured fungus clone 805 | 380 bp | Soil | / | Direct Submission by Beck, A. M. & Yannarell, A. C. |
| 111 | MF570575 | Uncultured fungus clone 2160 | 380 bp | Soil | / | Direct Submission by Beck, A. M. & Yannarell, A. C. |
| 112 | MF569442 | Uncultured fungus clone 835 | 379 bp | Soil | / | Direct Submission by Beck, A. M. & Yannarell, A. C. |
| 113 | MF570976 | Uncultured fungus clone 2646 | 382 bp | Soil | / | Direct Submission by Beck, A. M. & Yannarell, A. C. |
| 114 | MF570122 | Uncultured fungus clone 1620 | 378 bp | Soil | / | Direct Submission by Beck, A. M. & Yannarell, A. C. |
| 115 | MF569574 | Uncultured fungus clone 990 | 377 bp | Soil | / | Direct Submission by Beck, A. M. & Yannarell, A. C. |
| 116 | MF569170 | Uncultured fungus clone 524 | 379 bp | Soil | / | Direct Submission by Beck, A. M. & Yannarell, A. C. |
| 117 | MF569235 | Uncultured fungus clone 596 | 378 bp | Soil | / | Direct Submission by Beck, A. M. & Yannarell, A. C. |
| 118 | MF570795 | Uncultured fungus clone 2422 | 379 bp | Soil | / | Direct Submission by Beck, A. M. & Yannarell, A. C. |
| 119 | MF570099 | Uncultured fungus clone 1591 | 379 bp | Soil | / | Direct Submission by Beck, A. M. & Yannarell, A. C. |
| 120 | MF569930 | Uncultured fungus clone 1398 | 379 bp | Soil | / | Direct Submission by Beck, A. M. & Yannarell, A. C. |
| 121 | MF569964 | Uncultured fungus clone 1434 | 380 bp | Soil | / | Direct Submission by Beck, A. M. & Yannarell, A. C. |
| 122 | MF569734 | Uncultured fungus clone 1169 | 379 bp | Soil | / | Direct Submission by Beck, A. M. & Yannarell, A. C. |
| 123 | MF569726 | Uncultured fungus clone 1159 | 380 bp | Soil | / | Direct Submission by Beck, A. M. & Yannarell, A. C. |
| 124 | MF569973 | Uncultured fungus clone 1443 | 381 bp | Soil | / | Direct Submission by Beck, A. M. & Yannarell, A. C. |
| 125 | MF571286 | Uncultured fungus clone 3000 | 378 bp | Soil | / | Direct Submission by Beck, A. M. & Yannarell, A. C. |
| 126 | MF570515 | Uncultured fungus clone 2090 | 378 bp | Soil | / | Direct Submission by Beck, A. M. & Yannarell, A. C. |
| 127 | MF570398 | Uncultured fungus clone 1951 | 379 bp | Soil | / | Direct Submission by Beck, A. M. & Yannarell, A. C. |
| 128 | MF570025 | Uncultured fungus clone 1504 | 380 bp | Soil | / | Direct Submission by Beck, A. M. & Yannarell, A. C. |
| 129 | MF570666 | Uncultured fungus clone 2274 | 378 bp | Soil | / | Direct Submission by Beck, A. M. & Yannarell, A. C. |
| 130 | MF569897 | Uncultured fungus clone 1359 | 383 bp | Soil | / | Direct Submission by Beck, A. M. & Yannarell, A. C. |
| 131 | MF570734 | Uncultured fungus clone 2355 | 380 bp | Soil | / | Direct Submission by Beck, A. M. & Yannarell, A. C. |
| 132 | MF570742 | Uncultured fungus clone 2367 | 381 bp | Soil | / | Direct Submission by Beck, A. M. & Yannarell, A. C. |
| 133 | MF570521 | Uncultured fungus clone 2097 | 375 bp | Soil | / | Direct Submission by Beck, A. M. & Yannarell, A. C. |
| 134 | KX194514 | Uncultured fungus clone 2093 | 338 bp | Soil | / | Direct Submission by Beck, A. M. & Yannarell, A. C. |
| 135 | MF569388 | Uncultured fungus clone 776 | 376 bp | Soil | / | Direct Submission by Beck, A. M. & Yannarell, A. C. |
| 136 | MF570593 | Uncultured fungus clone 2182 | 375 bp | Soil | / | Direct Submission by Beck, A. M. & Yannarell, A. C. |
| 137 | MF571248 | Uncultured fungus clone 2960 | 378 bp | Soil | / | Direct Submission by Beck, A. M. & Yannarell, A. C. |
| 138 | MF570041 | Uncultured fungus clone 1526 | 375 bp | Soil | / | Direct Submission by Beck, A. M. & Yannarell, A. C. |
| 139 | MF570547 | Uncultured fungus clone 2125 | 376 bp | Soil | / | Direct Submission by Beck, A. M. & Yannarell, A. C. |
| 140 | MF569094 | Uncultured fungus clone 439 | 378 bp | Soil | / | Direct Submission by Beck, A. M. & Yannarell, A. C. |
| 141 | MF570310 | Uncultured fungus clone 1840 | 378 bp | Soil | / | Direct Submission by Beck, A. M. & Yannarell, A. C. |
| 142 | MF571249 | Uncultured fungus clone 2961 | 374 bp | Soil | / | Direct Submission by Beck, A. M. & Yannarell, A. C. |
| 143 | MF571226 | Uncultured fungus clone 2937 | 369 bp | Soil | / | Direct Submission by Beck, A. M. & Yannarell, A. C. |
| 144 | MF570148 | Uncultured fungus clone 1650 | 367 bp | Soil | / | Direct Submission by Beck, A. M. & Yannarell, A. C. |
| 145 | MF569775 | Uncultured fungus clone 1217 | 378 bp | Soil | / | Direct Submission by Beck, A. M. & Yannarell, A. C. |
| 146 | MF569730 | Uncultured fungus clone 1164 | 378 bp | Soil | / | Direct Submission by Beck, A. M. & Yannarell, A. C. |
| 147 | MF570418 | Uncultured fungus clone 1975 | 377 bp | Soil | / | Direct Submission by Beck, A. M. & Yannarell, A. C. |
| 148 | MF570392 | Uncultured fungus clone 1943 | 376 bp | Soil | / | Direct Submission by Beck, A. M. & Yannarell, A. C. |
| 149 | MF569731 | Uncultured fungus clone 1165 | 375 bp | Soil | / | Direct Submission by Beck, A. M. & Yannarell, A. C. |
| 150 | MF569012 | Uncultured fungus clone 349 | 375 bp | Soil | / | Direct Submission by Beck, A. M. & Yannarell, A. C. |
| 151 | KX193286 | Uncultured fungus clone 865 | 338 bp | Soil | / | Direct Submission by Beck, A. M. & Yannarell, A. C. |
| 152 | KY694749 | Uncultured fungus clone BS.DW2.5 | 340 bp | Rhizosphere | / | Direct Submission by Khan, A. L. |

*Acc. No.* accession number, *No.* number, */* no data available.

[1] Rosales-Castillo, J. A., Oyama, K., Vazquez-Garciduenas, M. S., Aguilar-Romero, R., Garcia-Oliva, F., & Vazquez-Marrufo, G. (2018). Fungal community and ligninolytic enzyme activities in quercus deserticola trel. litter from forest fragments with increasing levels of disturbance. *Forests*, 9(1), 11.

[2] Hu, W., Zhang, Q., Li, D., Cheng, G., Mu, J., Wu, Q., et al. (2014). Diversity and community structure of fungi through a permafrost core profile from the Qinghai-Tibet Plateau of China. *Journal of Basic Microbiology*, 54(12), 1331–1341.

[3] Bonito, G., Isikhuemhen, O. S., & Vilgalys, R. (2010). Identification of fungi associated with municipal compost using DNA-based techniques. *Bioresource Technology*, 101(3), 1.

[4] Zhao, Y., Li, W., Zhou, Z., Wang, L., Pan, Y., & Zhao, L. (2005). Dynamics of microbial community structure and cellulolytic activity in agricultural soil amended with two biofertilizers. *European Journal of Soil Biology*, 41(1-2), 21–29.

[5] Menkis, A., Marčiulynas, A., Gedminas, A., Lynikienė, J., & Povilaitienė, A. (2015). High-throughput sequencing reveals drastic changes in fungal communities in the phyllosphere of Norway spruce (*Picea abies*) following invasion of the spruce bud scale (*Physokermes piceae*). *Microbial ecology*, 70(4), 904–911.

[6] Hoellein,T.J., Tank,J., Kelly,J.J. & Rosi,E.J. (2010). Seasonal variation in nutrient limitation of microbial biofilms colonizing organic and inorganic substrata in streams. *Hydrobiologia*. 649. 331–345.

[7] Page, K. A., & Flannery, M. K. (2018). Chytrid fungi associated with pollen decomposition in crater lake, Oregon. *Fine Focus*, 4(1), 83–100.

[8] Menkis, A., Burokienė, D., Gaitnieks, T., Uotila, A., Johannesson, H., Rosling, A., et al. (2012). Occurrence and impact of the root-rot biocontrol agent *Phlebiopsis gigantea* on soil fungal communities in *Picea abies* forests of northern Europe. *FEMS microbiology ecology*, 81(2), 438–445.

[9] Kubartová, A., Ottosson, E., & Stenlid, J. (2015). Linking fungal communities to wood density loss after 12 years of log decay. *FEMS microbiology ecology*, 91(5).

[10] Hartmann, M., Lee, S., Hallam, S. J., & Mohn, W. W. (2009). Bacterial, archaeal and eukaryal community structures throughout soil horizons of harvested and naturally disturbed forest stands. *Environmental Microbiology*, 11(12), 3045–3062.

[a] Xing, B., Zhang, X. & Gong, J. Composition and variation of fungal communities in polluted estuarine sediments of the Laizhou Bay, northern China.

[b] Qin, M., Liu, Y. & Feng, H. Fungal community was significantly changed after twelve years of restoration in the Qinghai-Tibetan Plateau.

[c] Huang, Z. & Tan, Z. Internal transcribed spacers (ITS) DNA of eukarotype from the soil of ordinary banana farm.

[d] Zhao, Q. & Chen, H. Analysis of Endophytes Diversity in Wheat Seedlings by Metagenomic Technology.

[e] Lan, A. Culture-independent analysis for endophytic fungi diversity of Hanlian (*Magnolia soulangeana*).

[f] Priyamvada, H. High diversity of bioaerosols in the southern Indian region.

[g] Khan, A. L. & Asaf, S. Desert microbial community.

[h] Khan, A. L. Metagenomic of rhizosphere.

[i] Beck, A. M., Dawson, J. O. & Yannarell, A. C. Linkages between potential root-bound symbionts and non-symbiotic soil microbes in the alder and birch rhizosphere.
